# Bacterial Cellulose Aerogels Derived from Pineapple Peel Waste for the Adsorption of Dyes

**DOI:** 10.1021/acsomega.3c03130

**Published:** 2023-09-01

**Authors:** Ha Vu Le, Nghia Thi Dao, Ha Truc Bui, Phung Thi Kim Le, Kien Anh Le, An Thi Tuong Tran, Khoa Dang Nguyen, Hanh Huynh Mai Nguyen, Phuoc Hoang Ho

**Affiliations:** † Faculty of Chemical Engineering, Ho Chi Minh City University of Technology (HCMUT), 268 Ly Thuong Kiet Street, District 10, Ho Chi Minh City 740010, Viet Nam; ‡ Vietnam National University Ho Chi Minh City, Linh Trung Ward, Thu Duc District, Ho Chi Minh City 740010, Viet Nam; § Institute for Tropical Technology and Environmental Protection, 57A Truong Quoc Dung, Phu Nhuan District, Ho Chi Minh City 726500, Viet Nam; ∥ Chemical Engineering, Competence Centre for Catalysis, Chalmers University of Technology, Gothenburg SE-412 96, Sweden

## Abstract

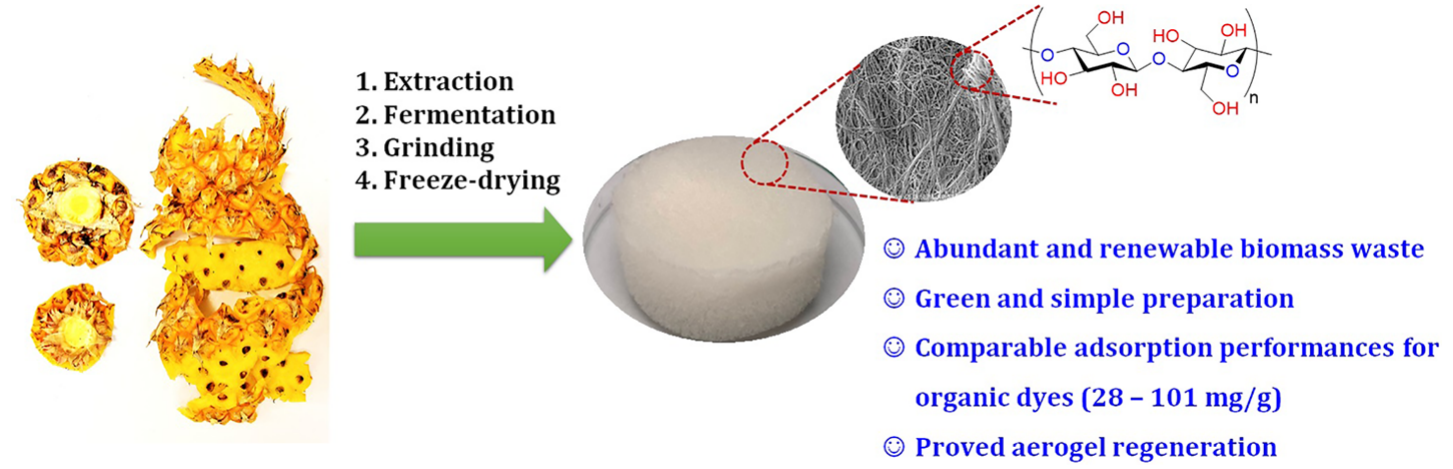

Valorization of pineapple peel waste is an attractive research topic because of the huge quantities of this byproduct generated from pineapple processing industries. In this study, the extract from pineapple waste was collected to produce a hydrogel-like form containing bacterial cellulose fibers with a three-dimensional structure and nanoscale diameter by the *Acetobacter xylinum* fermentation process. The bacterial cellulose suspension was subsequently activated by freeze-drying, affording lightweight aerogels as potential adsorbents in wastewater treatment, in particular the adsorptive removal of organic dyes. Intensive tests were carried out with the adsorption of methylene blue, a typical cationic dye, to investigate the influence of adsorption conditions (temperature, pH, initial dye concentration, time, and experiment scale) and aerogel-preparation parameters (grinding time and bacterial cellulose concentration). The bacterial cellulose-based aerogels exhibited high adsorption capacity not only for methylene blue but also for other cationic dyes, including malachite green, rhodamine B, and crystal violet (28–49 mg/g). However, its activity was limited for most of the anionic dyes, such as methyl orange, sunset yellow, and quinoline yellow, due to the repulsion of these anionic dyes with the aerogel surface, except for the case of congo red. It is also an anionic dye but has two amine groups providing a strong interaction with the hydroxyl group of the aerogel via hydrogen bonding. Indeed, the aerogel has a substantially large congo red-trapping capacity of 101 mg/g. Notably, the adsorption process exhibited similar performances, upscaling the solution volume to 50 times. The utilization of abundant agricultural waste in the simple aerogel preparation to produce a highly efficient and biodegradable adsorbent is the highlight of this work.

## 1 Introduction

Water plays a vital role in human life and human activities.^1,2^ However, explosive development in the manufacture of textiles, paints, leather, pharmaceuticals, and personal care products has resulted in the release of numerous organic toxins such as dyes, solvents, surfactants, and organometallic substances into the aqueous medium.^3−6^ Due to the carcinogenic, mutagenic, and allergenic effects of dyes and their increasing presence in various water resources, it is indeed necessary to thoroughly remove these toxic compounds from wastewater.^7^ Various methods have been suggested for the treatment of dye wastewater, including oxidation, coagulation, adsorption, irradiation, ion exchange, membrane separation, and biological processes.^8^ Among them, the adsorption method is favored because of its simplicity, high efficiency, and low cost.^9−12^ The discovery of efficient adsorbents plays an important role in adsorption technology. Moreover, from a sustainable development point of view, finding new environment-friendly porous biomaterials for capturing organic dyes from aqueous solutions is highly encouraged.^13^

Recently, aerogels have become known as a typical class of three-dimensional (3D) materials with a high degree of porosity and desirable properties for various applications, for example, air cleaning, water treatment, catalysis, or energy storage.^14,15^ These materials could be produced from various components such as silica, carbon, metal oxides, or organic polymers. However, the frameworks derived from polysaccharides, including cellulose, starch, chitosan, alginate, and pectin, have specifically drawn much attention because of their renewability as well as abundance in nature.^16^ Among these resources, bacterial cellulose (BC) exhibits interesting chemical and physical properties, including high crystallinity (>80%), a disordered 3D framework, good tensile strength, and moldability due to the formation of uniform nanofibers during the fermentation of carbohydrates by *A. xylinum*.^17,18^ Especially, in comparison with the utilization of plant cellulose, one of the most important benefits provided by bacterial cellulose is the absence of lignin and hemicellulose, which were removed under complicated and harsh conditions using hazardous chemicals.^19−21^ On the other hand, carbohydrate-containing agricultural commodities, e.g., coconut water and fruit extracts, have been widely used to fermentatively produce high-purity and low-cost cellulose on commercial scales for well-known applications in food and pharmaceuticals.^22−28^ Based on such outstanding features, bacterial cellulose is considered as a promising renewable material for yielding functional aerogels toward removing harmful contaminants such as organic dyes and heavy-metal cations from aqueous solutions.^18^

In this work, pineapple peel waste collected from pineapple-based food manufacturing processes was employed as an abundant carbohydrate source to produce bacterial cellulose via fermentation at ambient conditions. The bacterial cellulose was subsequently transformed into lightweight aerogels using a freeze-drying method. The characterizations of BC-based aerogels were performed by different techniques, including powder X-ray diffraction (PXRD), thermogravimetric analysis (TGA), nitrogen physisorption at 77 K, and scanning electron microscopy (SEM), to confirm the non-ordered and porous 3D structure of the bacterial cellulose network in the aerogels. These biomaterials were applied as green and inexpensive adsorbents for the removal of organic dyes from water, showing promising performances without any further chemical modification.

## 2 Material and Methods

All chemicals were purchased from Sigma-Aldrich and Acros and used as received without any further purification.

### 2.1 Synthesis of Aerogel

**Figure 1:**
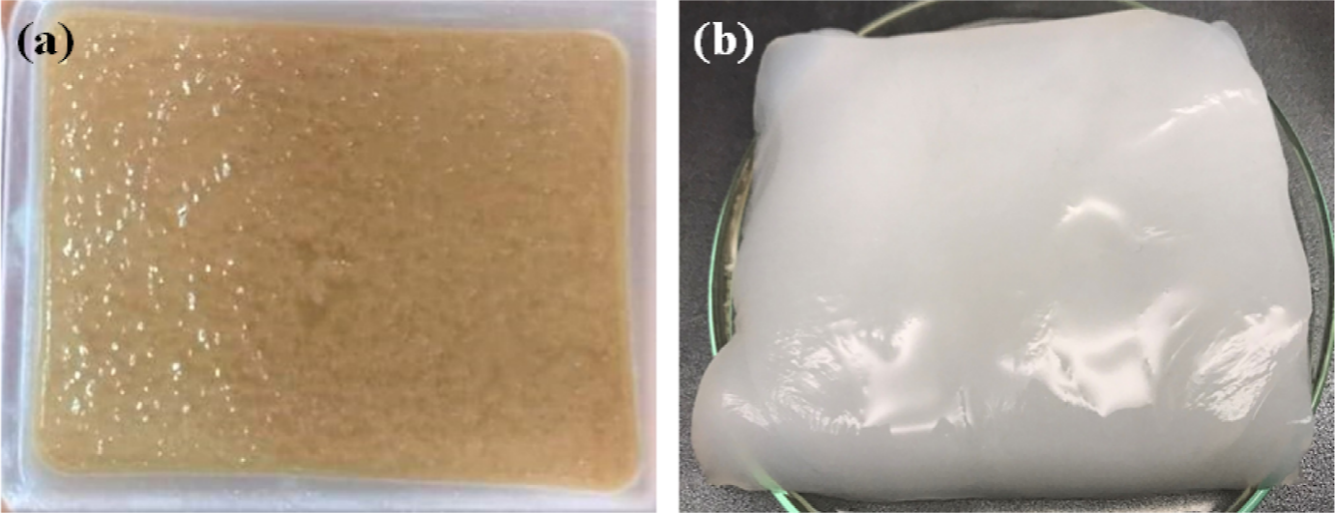
Photographs of (a) 100-g batch of the nata de pina preparation via the fermentation of the extract of pineapple peel waste and (b) *nata de pina* after washing with NaOH and water, sequentially.

The extract of pineapple peel waste (*Ananas comosusSpanish*, planted in Long An, Viet Nam) was collected, filtrated, and fermented by *A. xylinum* under aerobic conditions.^29^ After 7 days, a hydrogel-like layer with a thickness of approx. 2 cm formed on the surface of the fermentative phase, which is commonly known as *nata de pina*, was collected. Raw *nata de pina* (Figure 1a) was immersed in a sodium hydroxide solution (1 M) for 12 h and subsequently washed with water until neutralization (Figure 1b).

Prior to further experiments, the washed nata de pina samples were cut into approx. one-centimeter cubes. The cellulose content was determined to be approx. 0.80 wt % based on drying the washed sample at 120 °C for 24 h. Nata de pina pieces were mixed with water in a 1:1 ratio. The mixture was subsequently ground using a Philips HR2531 hand blender at 650 W for different time intervals (1–5 min) and added with water to obtain a suspension with a predetermined BC content (0.24 to 0.80 wt %). The obtained mixtures were then subsequently frozen at −20 °C for 24 h. The bacterial cellulose aerogels were obtained by freeze-drying the frozen samples at 0.5 mbar for 48 h using a Toption TPV-50F vacuum freeze dryer to produce cylinder-shaped aerogel samples with a typical diameter of approx. 6.5 cm (d) and thickness of approx. 3.0 cm (h).

Bulk density of the collected aerogel samples was determined via the equation ρ_a_ =4·*m*/π × *d*^2^ × *h* in which *m*, *d*, and *h* are, respectively, the weight, diameter, and thickness of the samples.

Porosity of aerogel samples was calculated via the equation Ø = 100 × (1 – ρ_a/_ρ_b_) in which ρ_a_ is the bulk of cellulose aerogels (g/cm^3^) and ρ_b_ is the crystalline cellulose density (1.5 g/cm^3^).^30−32^

### 2.2 Adsorption Study

Adsorption capacity of aerogel was investigated for two groups of organic dyes, including cationic dyes (methylene blue, rhodamine B, crystal violet, and malachite green) and anionic dyes (methyl orange, quinoline yellow, sunset yellow, and congo red) (Table 1). In a typical experiment, approx. 20 mg of the BC aerogel was loaded into 10 mL of a solution containing 50 ppm of methylene blue (MB). The adsorption was performed at 30 °C under vigorous stirring for 30 min. The aerogel was subsequently separated from the solution by filtration. The MB concentrations in the solution before and after the adsorption course were determined by ultraviolet–visible (UV–vis) spectroscopy using a G10S UV–vis device (Thermo Scientific, Waltham, Massachusetts, USA). Additionally, the experiments for the MB adsorption and the aerogel preparation were also performed at different conditions to study the effect of temperature (20–60 °C), pH (3.6–11.0), initial dye concentration (0–400 ppm), adsorption time (0–120 min), bacterial cellulose content (0.24–0.80 wt.%), grinding time (1–5 min), and experiment scale (10–500 mL) on the MB trapping efficiency. The error bar for each data point was the standard deviation from three experiments.

**Table 1:**
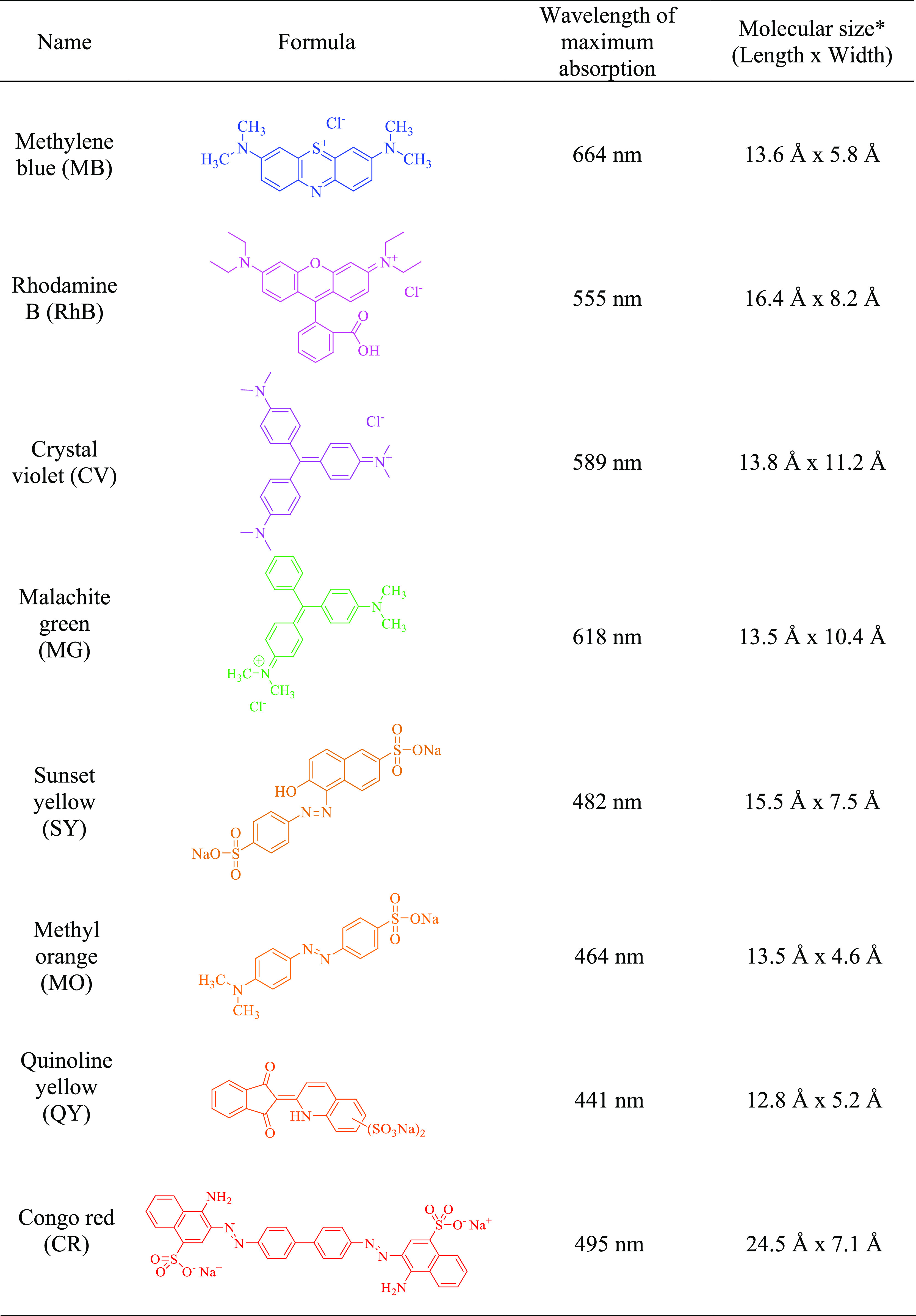
Details of Organic Dyes Used in This Study

* Sizes of the organic dye molecules (length x width) were estimated by the Materials Studio program.

Two models including pseudo-first-order and pseudo-second-order equations (eqs 1 and 2) were applied to study the kinetics of the MB adsorption by the BC aerogel.^33^
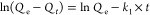
1
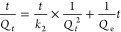
2in which *Q*_e_ is the adsorption capacity at the equilibrium (mg/g), *Q*_*t*_ is the adsorption capacity at a given time (mg/g), *k*_1_ and *k*_2_ are the rate constants of the adsorption models, respectively.^33^

The interaction of the MB molecules with the BC surface was also investigated by employing the Langmuir and Freundlich adsorption isotherm equations (eqs 3 and 4).^34^
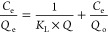
3
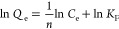
4in which *Q*_e_ is the adsorption capacity at the equilibrium (mg/g), *C*_e_ is the equilibrium concentration in solution (mg/L, ppm), *Q*_o_ is the maximum amount of adsorption (mg/g), and *K*_L_ and *K*_F_ are the Langmuir and Freundlich constants, respectively.^34^

For the recycling test, the aerogel was collected from a 500 mL-scale experiment by gravity filtration before being washed with acetone containing 5 wt % of HCl five times to remove MB. The resulting bacterial cellulose phase was subsequently washed with water until neutralization. This washed bacterial cellulose suspension was frozen at −20 °C for 24 h and freeze-dried, yielding the regenerated aerogel for the next adsorption experiment under identical conditions.

### 2.3 Material Characterization

Textural properties of the cellulose materials were determined by nitrogen physisorption at 77 K using an ASAP 2020 instrument (Micromeritics, USA). Before each analysis, samples were degassed at 60 °C under vacuum for 12 h. The specific surface area was determined over a relative pressure range of *P*/*P*_o_ = 0.05–0.30 using the Brunauer–Emmett–Teller (BET) method.

PXRD patterns were obtained on a D8 ADVANCE diffractometer (Bruker AXS, Germany) using Ni-filtered Cu Kα radiation. Each measurement was performed in a 2θ range of 10–70° with an angular step size of 0.0105° and a scanning rate of 0.63° per min.

Morphology of the aerogel samples was recorded by field-emission SEM (FESEM) using a Hitachi S-4800 microscope with a magnification of 25,000× at an accelerating voltage of 10 kV.

Thermal stability of cellulose was analyzed by TGA on a TA Instruments SDT Q600 thermal gravimetric analyzer. Approx. 10 mg of the aerogel sample was placed into an alumina pan, and the sample was heated from 30 to 900 °C at a rate of 10 °C/min in air.

Average size of the nata de pina suspension mixture after grinding was determined by laser diffraction spectroscopy using a Horiba LA-950V2 laser diffraction spectrometry.

Fourier transform infrared spectroscopy (FT-IR) measurement was performed using a Bruker Vertex 70 spectrometer for the aerogel sample dispersed on a potassium bromide pellet. Each measurement was accumulated from 32 scans at a resolution of 4 cm^–1^ recorded in the 4000–500 cm^–1^ range.

The point of zero charge (pH_PZC_) of the BC aerogel material derived was determined by monitoring the pH change of a 1.0 mol/L NaCl solution with varied pH.^35^ In detail, the pH value (pH_1_) of the initial NaCl solution was predetermined in the range of 3–11 by adding a 5 wt % solution of HCl or NaOH. 20 mg of the aerogel was subsequently added to the obtained NaCl solution. After 24 h under vigorous stirring, the solution pH (pH_2_) was measured again. The pH difference (ΔpH = pH_2_–pH_2_) was plotted versus pH_1_, and pH_PZC_ was the vertical projection of the curve.

## 3 Results and Discussion

### 3.1 Material Characterization

**Figure 2:**
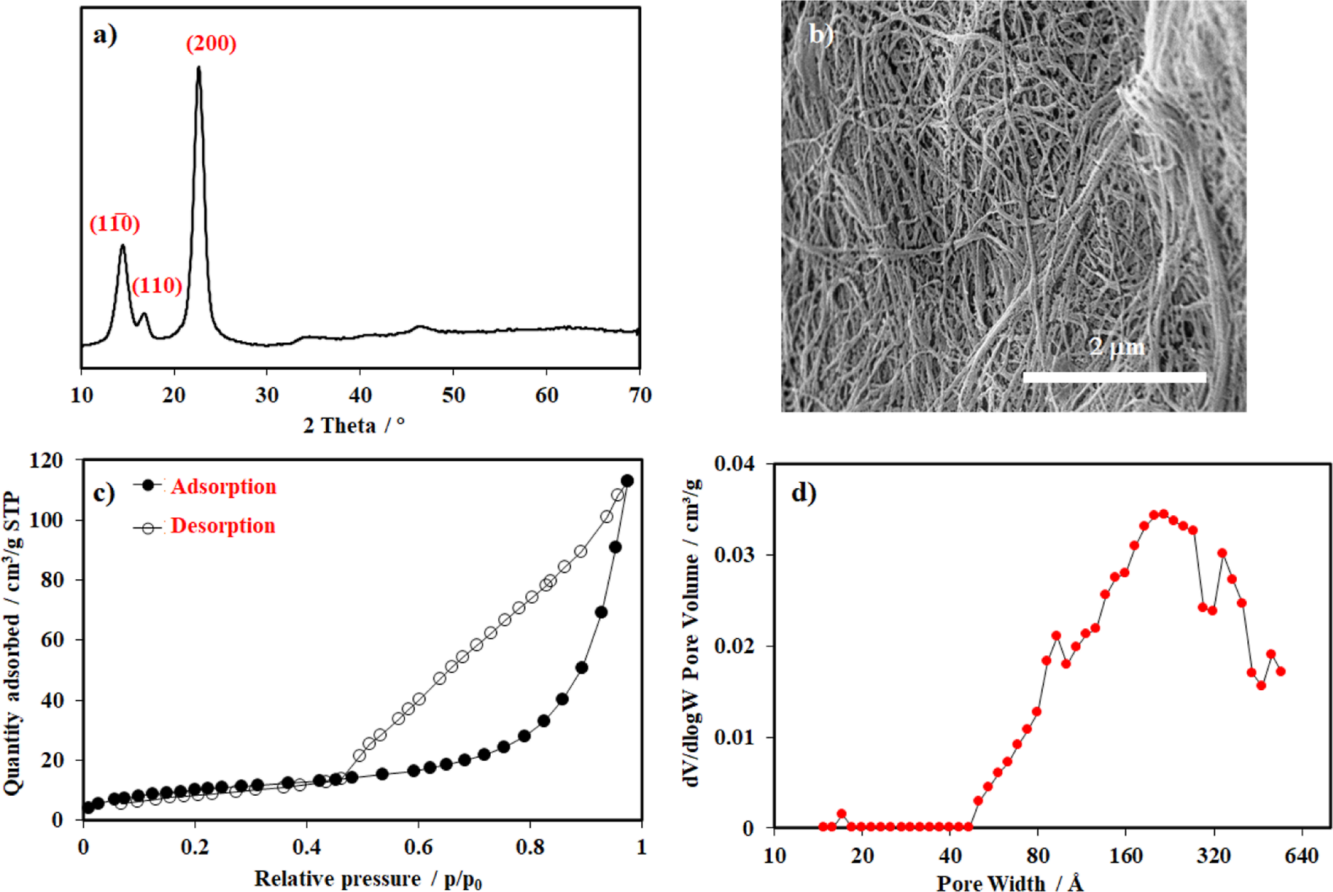
Characterizations of the aerogel material derived from pineapple peel waste: PXRD pattern (a); SEM image (b); N_2_ physisorption isotherm (c); and pore size distribution (d).

In this study, *nata de pina* containing approx. 0.80 wt % of bacterial cellulose was produced from the pineapple peel extract for many different batches (approx. 100 g of *nata de pina* per batch) without the addition of any further nutrients and carbohydrate resources. The PXRD pattern of the BC aerogel material prepared from the *nata de pina* hydrogel showed featured reflections of crystalline cellulose at 2θ = 22.8, 14.6, and 17.7°, which were assigned to the planes of (200), (11̅0), and (110), respectively (Figure 2a). Notably, these intense peaks also demonstrated the high crystalline nature of the aerogel derived from bacterial cellulose, which was consistent with previous studies on cellulose obtained from bacterial fermentation.^29,36,37^ The morphology of the aerogel sample was also observed through the SEM analysis (Figure 2b). The bacterial cellulose material consisted of fibers and bundles with diameters ranging from 20 to 50 nm. These bundles were irregularly arranged with a high degree of interconnection to form the 3D matrix due to the accidental appearances and movements of *A. xylinum* bacteria in the fermentation medium.^38^

The porosity of the bacterial cellulose network was strongly dependent on the drying method for the removal of solvents, as previously reported in the literature.^39,40^ In fact, a non-porous thin sheet was produced by employing thermal drying under atmospheric or reduced pressure to treat the hydrogel form of bacterial cellulose. Under such traditional drying approaches, water vapor was diffused throughout the cellulose matrix, leading to agglomeration of the fibers. In addition, abundant hydrogen bonding in the bacterial cellulose network could also accelerate the structural collapse.^40−43^ Alternatively, freeze-drying and supercritical CO_2_ drying have been commonly used for the preparation of aerogels from bacterial cellulose due to the good preservation of the pristine 3D network.^43−46^ Typically, using these as-prepared bacterial cellulose sources at a concentration of 0.40% in a two-minute ground suspension with water, the freeze-dried bacterial cellulose aerogel could be obtained with an average bulk density of 0.0051 ± 0.0003 g/cm^3^ which was determined based on 9 different samples. As expected, the aerogel presented a BET surface area of approx. 35 m^2^/g. The obtained isotherm for this sample can be classified as Type II according to the IUPAC classification for macroporous materials. However, a hysteresis loop at the relative pressure from 0.80 to 0.96 proved the appearance of both mesopores and macropores in the aerogel (Figure 2c).^47−50^ The DFT-based pore size distribution indeed showed a wide range of pore sizes larger than 50 Å, which was attributed to the 3D bacterial cellulose network generated in nata de pina (Figure 2d).^40^ Furthermore, FT-IR measurement was performed to identify the functional groups present in the aerogel (Figure S1). Accordingly, three characteristic stretching vibrations typically assigned to hydroxyl (O–H), methylene (−CH_2_−), and ether groups (C–O) of cellulose were, respectively, observed at 3342, 2894, and 1056 cm^–1^, respectively.^51,52^ These electron-rich saturated oxygen atoms could attract cationic species in the aqueous phase. Meanwhile, the strong polarization of the hydroxyl group offers a potential platform for the formation of hydrogen bonds with the electronegative atoms in the dye molecules. Such electrostatic interactions could be responsible for the aqueous adsorption of organic dyes onto the aerogel surface.

**Figure 3:**
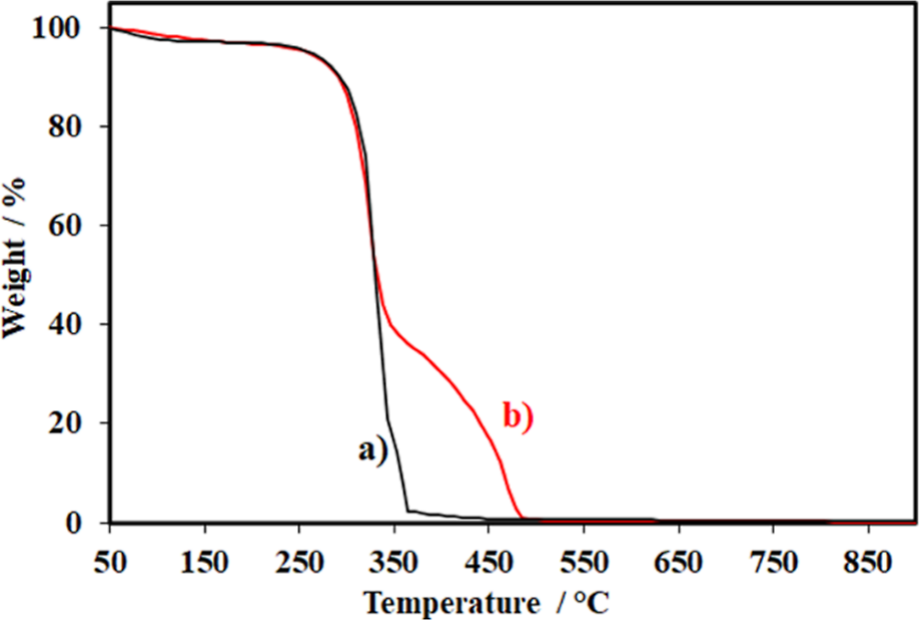
TGA profiles of the fresh BC aerogel (a) and the BC sample after the adsorption experiment with a 50-ppm solution of MB for 30 min (b).

The thermal stability of the bacterial cellulose aerogel prepared by freeze-drying at 0.5 mbar for 48 h was investigated via TGA. The profile was recorded from 30 to 900 °C under an air atmosphere. A minor weight loss of approx. 5% was observed up to 300 °C due to the release of moisture in the sample. Above 300 °C, the fresh BC sample was decomposed and combusted rapidly into CO_x_ and H_2_O, and the combustion was almost completed at 360 °C (Figure 3a).^53^ The TGA analysis was also employed to discover the thermal behavior of the aerogel sample after the adsorption experiment with a 50-ppm solution of methylene blue for 30 min. It was observed that this TGA profile almost overlapped with that of the bare aerogel up to 300 °C, indicating a reassembling thermal behavior of the BC aerogel in both samples. However, a distinct difference in weight loss was observed for the samples from 300 to 490 °C. The slow rate of weight loss in the profile of the used aerogel sample was associated with the presence of methylene blue trapped in this adsorbent after the adsorption (Figure 3b). To further clarify the capture efficiency of the bacterial cellulose aerogel derived from pineapple peel waste for methylene blue, a series of experiments were carried out based on variations of the adsorption conditions including exposure time, initial concentration, temperature, and initial pH value of the methylene blue solution.

### 3.2 Adsorption Studies

**Figure 4:**
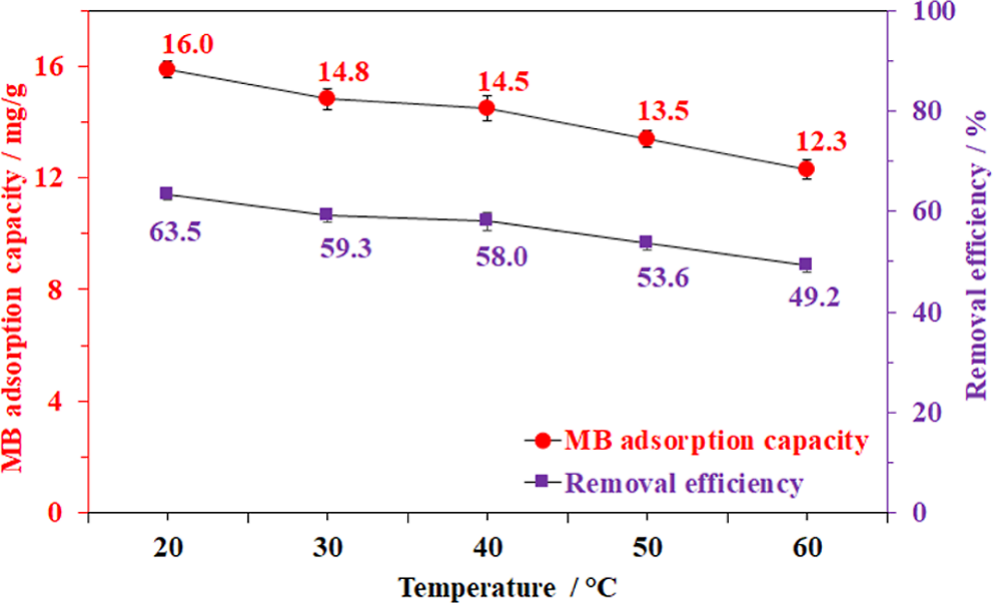
Effect of temperature on methylene blue adsorption capacity. Adsorption conditions: 20 mg of aerogel, 10 mL of 50 ppm MB solution at pH = 6.5 for 30 min.

First, the MB adsorption was carried out at different temperatures for 30 min to investigate its influence on the performance of the prepared material in trapping MB from the aqueous solution. As shown in Figure 4, the MB trapping capacity could reach up to 16 mg/g, corresponding to a removal efficiency (RE) of approximately 63.5% at 60 °C, and this value gradually decreased to 12.3 mg/g (RE ≈ 49.2%) as the adsorption process was carried out at 20 °C. This drop implied that capturing methylene blue from an aqueous solution by the BC aerogel derived from pineapple peel waste was an exothermic process. This could be caused by the formation of physical or chemical bonds between the active sites and adsorbates.^54−56^

**Figure 5:**
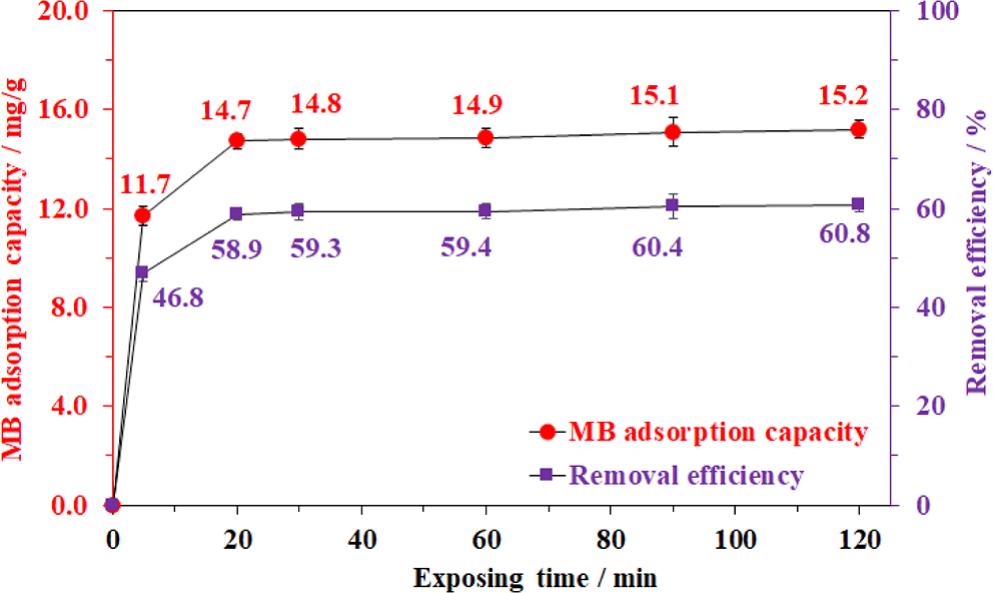
Effect of adsorption time on methylene blue adsorption capacity. Adsorption conditions: 20 mg of aerogel, 10 mL of 50 ppm MB solution at 30 °C, and pH = 6.5.

**Figure 6:**
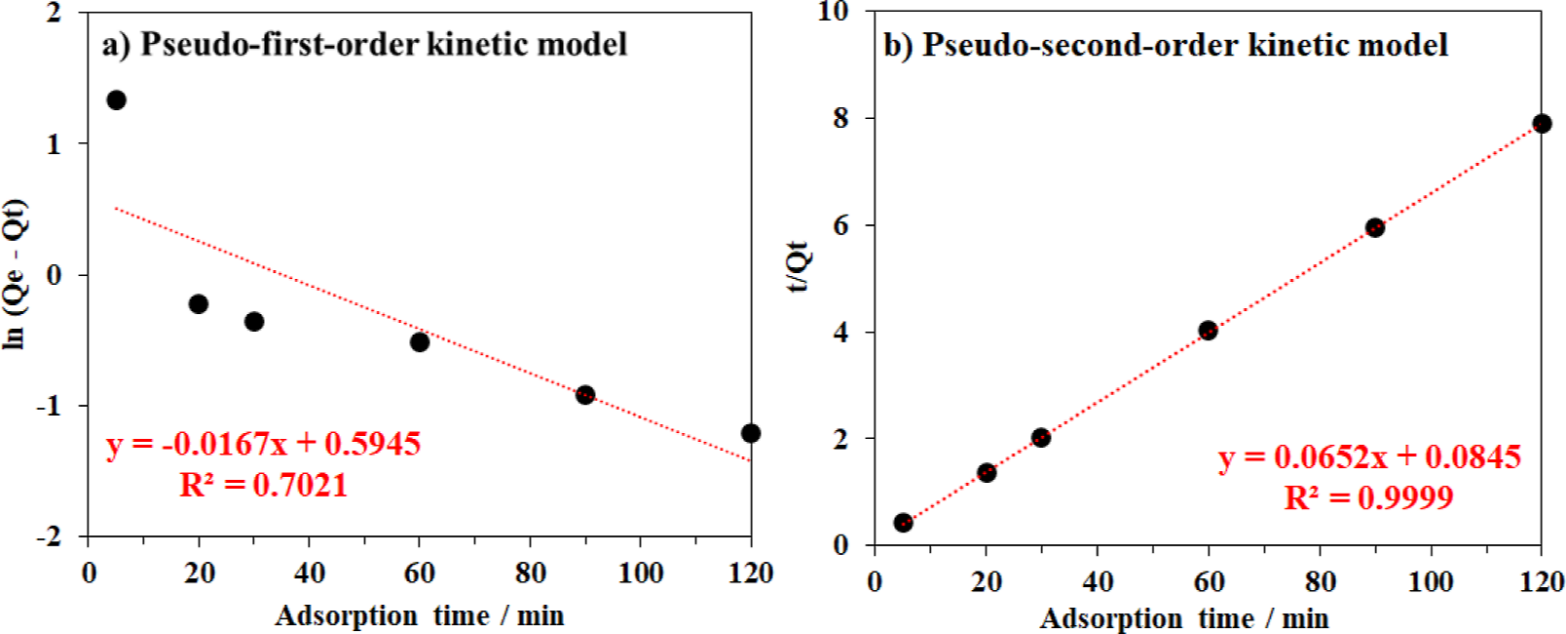
(a) First-order and (b) second-order adsorption kinetics of the methylene blue adsorption using the bacterial cellulose aerogel material.

**Figure 7:**
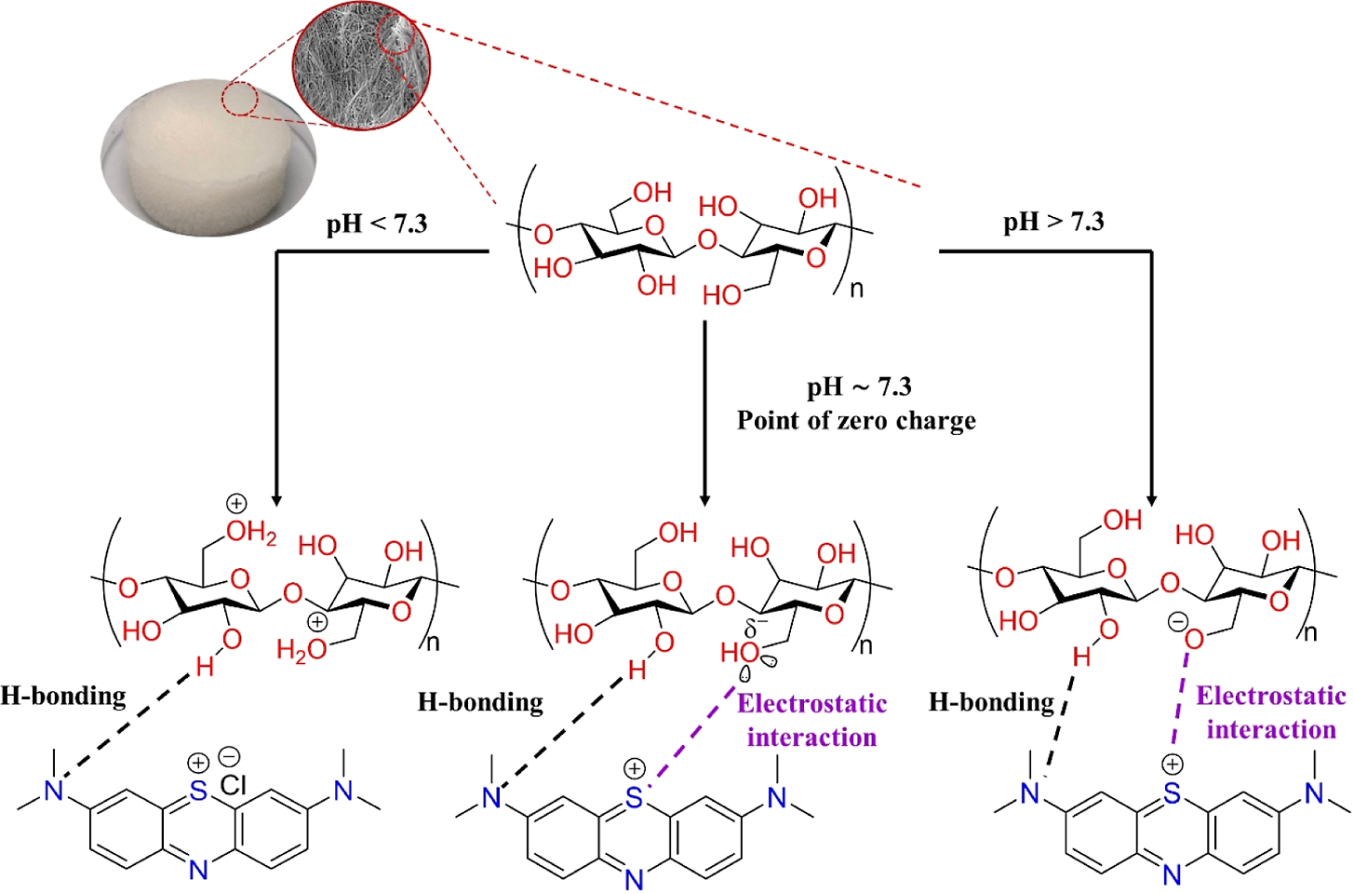
Proposal of adsorptive sites and their interactions with methylene blue molecules at different pH values.

In the next study, the effect of the adsorption time on the performance of the prepared aerogel was also investigated at various time intervals from 0 to 120 min at 30 °C (Figure 5). The trapping capacity rapidly increased to 14.7 mg/g (RE ≈ 58.9%) in the first 20 min of the adsorption, then the removal efficiency steadily rose and reached a saturation state after about 30 min. To clarify the adsorption kinetics of MB molecules into the cellulose framework, this time-dependent adsorption profile was further employed based on the pseudo-first-order and pseudo-second-order models (eqs 1 and 2).^33^ The correlation coefficients were found to be 0.999 and 0.702 for the second-order and the first-order models, respectively (Figure 6), implying that the adsorption of MB on cellulose aerogel was better fitted with the pseudo-second-order model. Based on the previous reports, the rate-determining step in this adsorption process was related to both physical and chemical interactions, which could be attributed to the functional group present in the aerogel matrix and the MB molecule. Namely, the bacterial cellulose framework includes a large number of electron-rich saturated oxygen atoms in the hydroxyl (−OH) and ether (−O−) groups, while the cation in MB is not only positively charged but also possesses many nitrogen atoms with lone electron pairs.^33,52,57,58^ Therefore, the adsorption of MB into the BC network could be assigned to different electrostatic interactions, i.e., the attraction force between the saturated oxygen atoms in cellulose and the cationic species and the hydrogen bonding of the hydroxyl hydrogen atoms with the nitrogen atoms in MB (Figure 7).^59−61^

**Figure 8:**
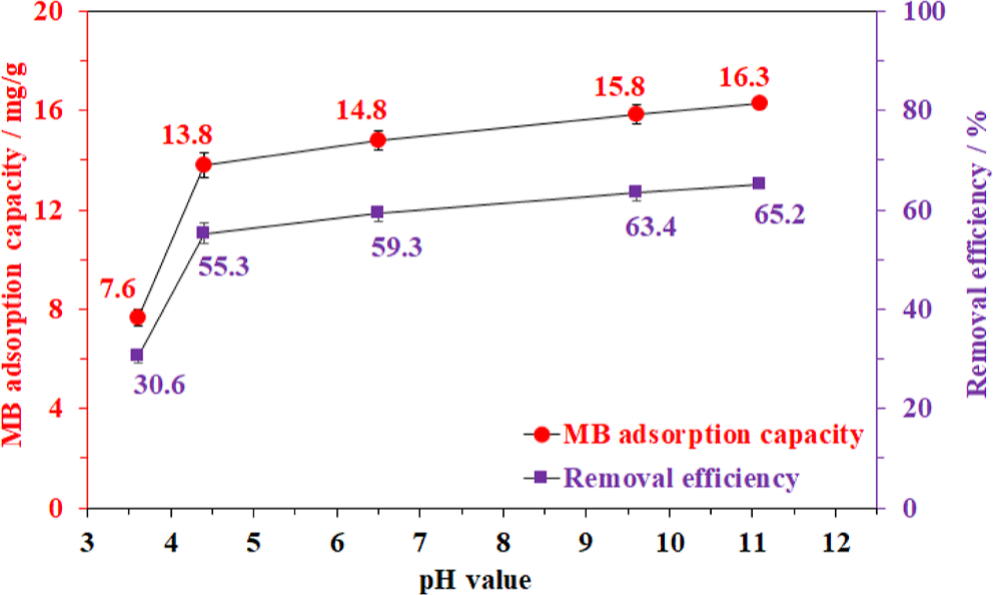
Effect of the pH value on methylene blue adsorption capacity. Adsorption conditions: 20 mg of aerogel, 10 mL of 50 ppm MB solution at 30 °C for 30 min.

Since the adsorption process was based on electrostatic interactions, the initial pH of the aqueous solution might play an important role in the MB-trapping efficiency of the BC aerogel.^9^ In other words, the removal of methylene blue could be impacted by the presence of guest ions, such as proton, hydroxide, or other charged species.^10,62^ Therefore, the adsorption of methylene blue was then studied in the range of solution pH from 3 to 11. The result showed that increasing the pH was beneficial for the trapping of MB into the aerogel material (Figure 8). Particularly, the experiment at the initial pH of 6.5 afforded an adsorption capacity of approx. 14.8 mg/g (RE = 59.3%). The MB uptake was significantly reduced by half as the solution was further acidified to pH = 3.6. In contrast, only a minor activity enhancement of 10% occurred as the pH increased from 6.5 to 11.

To elucidate the pH-dependent adsorptive behavior of the aerogel, its zero-charge point (pH_pzc_) was also determined via the pH alteration in various solutions added with the aerogel. As shown in Figure S2, the entire surface charge of the cellulose material adsorbent is equal to zero at pH ≈ 7.3, close to the results previously obtained for the pure cellulose-based materials.^51,63^ Therefore, the aerogel surface should be more positively charged under acidic conditions because the electron-rich oxygen atoms on cellulose chains would be facilely occupied by the H^+^ ions. Notably, Leyva-Ramos and co-workers previously described that methylene blue molecules exist in an aqueous solution as both cationic species and undissociated molecules. Its neutral form could occupy approx. 75% at pH = 3.6, while the cationic one appeared under less acidic conditions and could be the sole form at pH > 6.^64^ Obviously, the access of the MB cationic species to the cellulose network might be significantly hindered due to the repulsive interaction between the same charges at the very low pH of 3.6. However, a minor amount of MB could be adsorbed under this condition, possibly based on the hydrogen bonding between nitrogen in MB and hydrogen of the unprotonated hydroxyl groups (Figure 7a).^10,55^ In contrast, the solution basification induced the deprotonation of the hydroxyl groups, increasing the negative charge density of the aerogel surface. As a result, these negatively charged functional groups would strongly interact with the positively charged MB species (the dominant form of MB at pH > 6) via the proposed electrostatic interactions (Figure 7c). It should be noted that the deprotonation was likely insignificant under the applied conditions; therefore, only slight improvements in the MB-trapping efficiency were observed based on the pH increase.^65,66^

**Figure 9:**
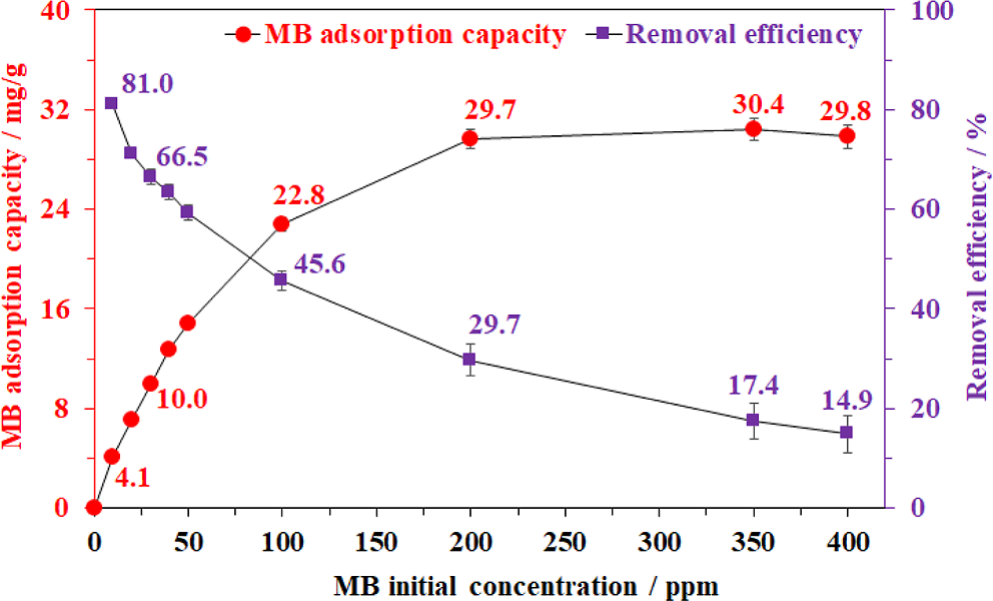
Effect of initial methylene blue concentration on adsorption capacity. Adsorption condition: 20 mg of aerogel, 10 mL of MB solution at 30 °C for 30 min and pH = 6.5.

Varying the initial methylene blue concentration for the adsorption course in the range of 0–400 ppm was also conducted to determine the maximal adsorption capacity of this BC aerogel (Figure 9). The adsorption capacity for MB considerably increased from 4.1 to 29.7 mg/g with growth in the initial concentration of MB from 10 to 200 ppm, respectively. No further improvements in the removal capacity were observed for higher concentrations. As the solutions become more concentrated, the ratio of the adsorbates over open adsorptive centers increases, providing a stronger driving force for the adsorption of MB.^62^ In contrast, in terms of the removal efficiency, 81% of MB was trapped by the BC aerogel at the low MB concentration, namely 10 ppm. Increasing the initial MB concentration led to a corresponding decrease in the MB removal percentage due to the MB excess in the solution. However, this progress was also controlled by the number of adsorptive sites. In other words, the adsorption efficiency of aerogel would reach the saturation state after all active centers, namely the hydroxyl groups and the rich-electron oxygen atoms on the cellulose backbone, were occupied by the methylene blue cations.^10,67^ Therefore, concentrations higher than 200 ppm cannot provide more dynamics to further improve MB trapping.

**Figure 10:**
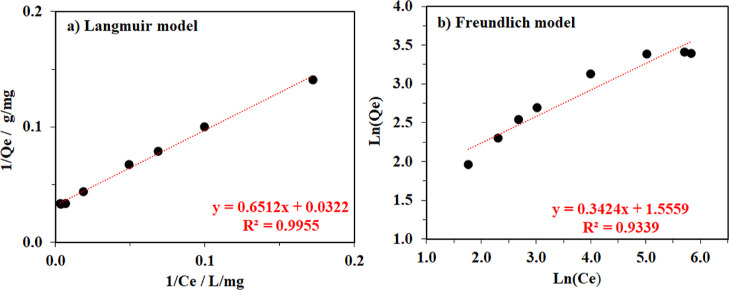
MB adsorption isotherms of the BC aerogel derived from pineapple peel waste fitted by Langmuir model (a) and Freundlich model (b).

These initial concentration-dependent adsorption results were employed to continuously investigate the adsorption mechanism of the MB on the cellulose surface using the Langmuir and Freundlich equations (eqs 3 and 4). In detail, the Langmuir model assumes monolayer coverage of adsorbate onto a homogeneous adsorbent, while the Freundlich one hypothesizes reversible adsorption including both formations of monolayer and multilayer of adsorbates.^55,57,68^ It was observed that the MB adsorption isotherm data of aerogel material derived from pineapple peel waste fit better to the Langmuir model with a high correlation coefficient of 0.996, than the Freundlich equation which had a low correlation coefficient of 0.934 (Figure 10). In addition, the maximal adsorption capacity for MB determined by the Langmuir model was approx. 31.1 mg/g, very close to the experimental result recorded at the high MB concentrations. In other words, the adsorption of MB molecules likely carried out and generated one adsorbate molecular layer onto the fixed active sites of cellulose fibers. And, there was no interaction between the adsorbed molecules, including neighboring sites.^68,69^

**Figure 11:**
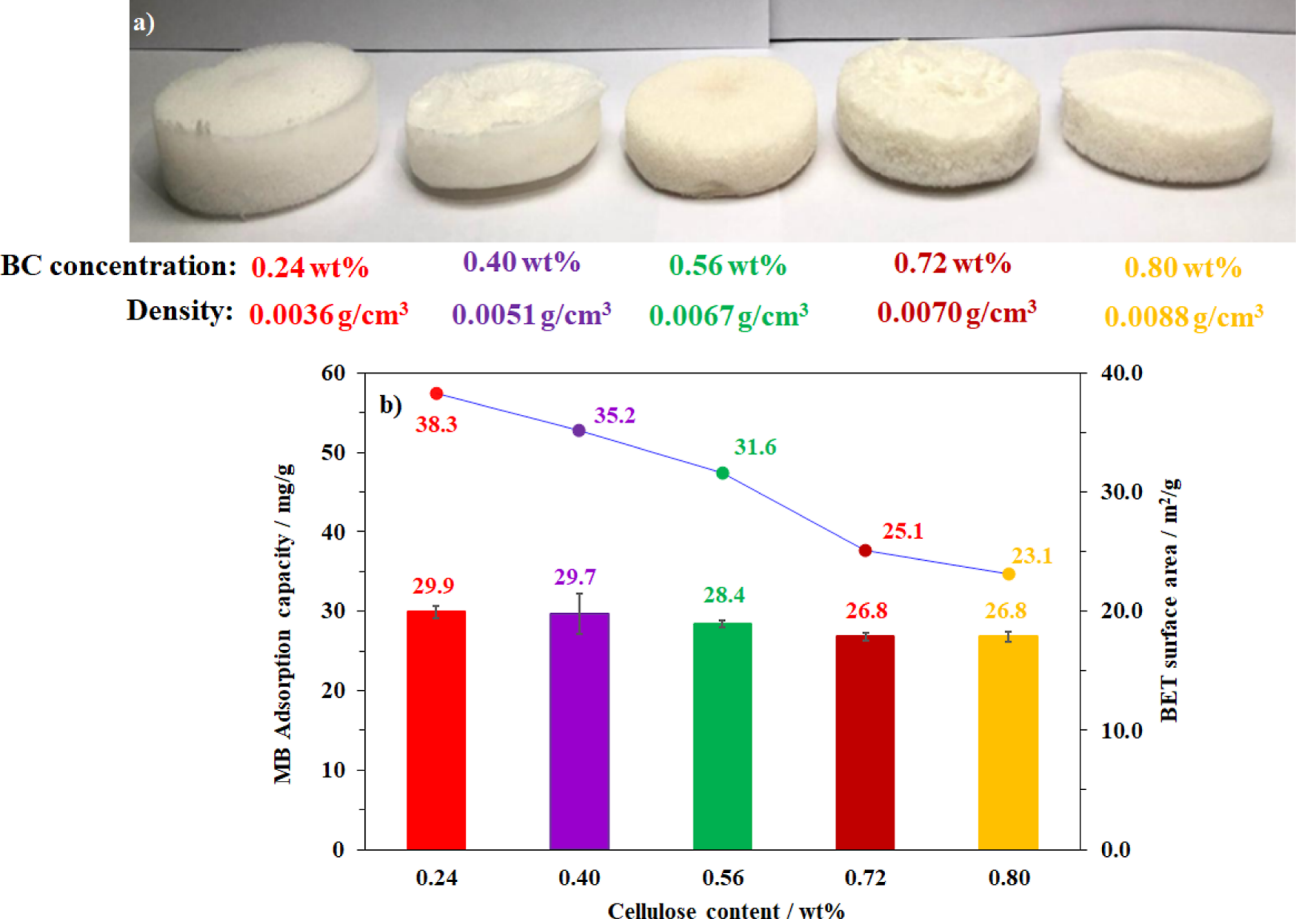
Photographs of aerogel samples prepared from the suspensions with varied BC concentrations (a) and their effect on the adsorption capacity and the specific surface area of the obtained aerogels (b).

The aerogel preparation conditions also impacted the MB adsorption efficiency due to their effect on the formation of the BC matrix. Accordingly, different concentrations of BC in water from 0.24 to 0.80 wt % were prepared after 2 min grinding. Subsequently, freeze-drying was carried out, yielding five corresponding aerogel samples. As shown in Figure 11a, increasing the cellulose content in the suspension phase led to significant increases in the as-prepared aerogel density; however, large drops (up to 40%) in the surface area were observed. Interestingly, only minor losses (1–10%) in the adsorption efficiency of the BC aerogel were recorded, indicating a weak correlation between the aerogel’s performance and its porosity (Figure 11b). It was based on the fact that BC aerogels can undergo hydration in the aqueous phase, thereby further changing the pristine aerogel structure.

**Figure 12:**
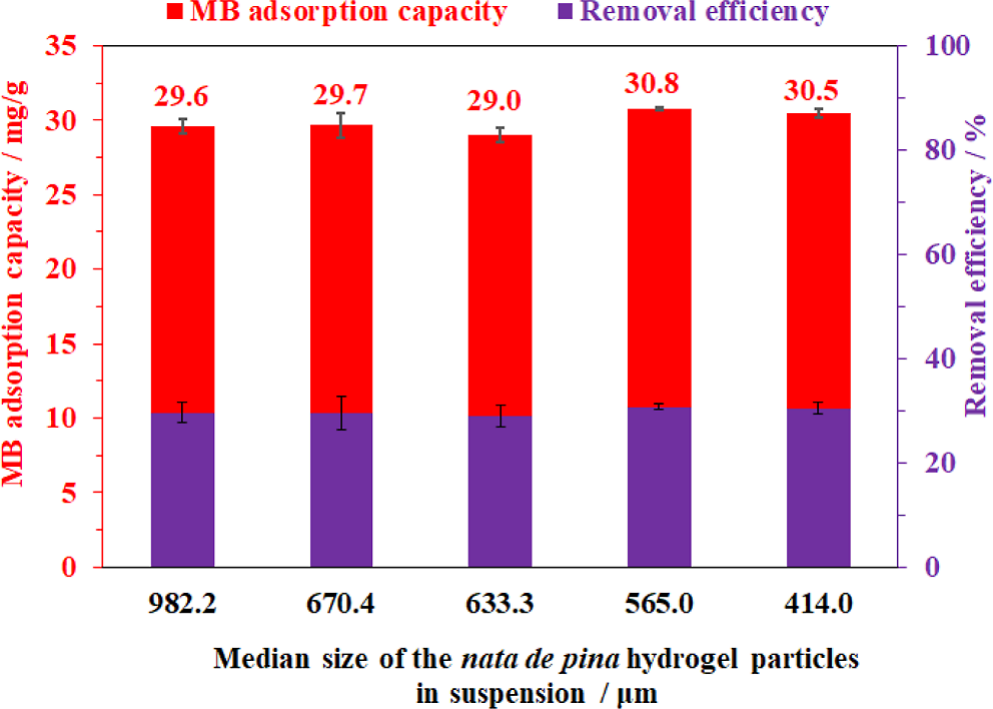
Methylene blue adsorption capacity on the aerogel samples prepared from the suspensions with different sizes of *nata de pina* hydrogel particles. Adsorption condition: 20 mg of aerogel, 10 mL of MB solution with a concentration of 200 ppm, at 30 °C for 30 min and pH = 6.5.

Furthermore, the effect of the *nata de pina* size on the aerogel texture and its adsorption performance was investigated. In detail, the grinding time of the *nata de pina*/water mixture was varied from 1 to 5 min to obtain different particle sizes of *nata de pina* before the suspension phases were prepared at the same BC concentration of 0.40 wt % for freeze drying. This provided another series of five BC aerogel samples. It should be observed that grinding was essential to obtain the BC aerogel. Freeze-drying the pristine *nata de pina* sample in 1 cm cubes indeed yielded thin sheets with negligible porosity and adsorption capacity for MB. The BC frameworks in this form needed to be fragmented and interleaved with others by grinding, which was followed by freeze-drying, affording a desired aerogel structure. As can be predicted, prolonging grinding considerably reduced the *nata de pina* size; however, the density of the obtained BC aerogels remained almost unchanged with no significant alterations detected (Table 2), proving that the obtained aerogel texture was independent of the *nata de pina* size within the studied time range of grinding. Therefore, the MB trapping capacities were recorded at approx. 30 mg/g for all of the samples observed (Figure 12).

**Table 2:** Bacterial Cellulose Aerogels Prepared From the Suspensions With Different Grinding Times

Grinding time (min)	1	2	3	4	5
Median size of *nata de pina* (μm)	982.2	670.4	633.3	565.0	414.0
Density of the obtained aerogel (g/cm^3^)	0.0051	0.0051	0.0050	0.0057	0.0052

In the last two decades, various plant-based cellulose waste sources, such as bagasse, sawdust, sugar beet pulp, cotton, and sago pith, have been recycled for the adsorption of MB. In general, complicated chemical treatments were required for the removal of lignin and other impurities which can affect the textural properties of the desired adsorbent as well as prevent the functional groups on the cellulose fiber from further chemical modification or interaction with the adsorbate species. Using the adsorbent derived from plant cellulose, the MB uptake was reported to be in a broad range of 1.4–42.2 mg/g, depending on the material type and composition and adsorption conditions (entries 1–10, Table 3). On the other hand, modification of the cellulose surface and combination with other materials have recently allowed significant improvements in the adsorption activity (Entries 11–14, Table 3). Although a high adsorption capacity could be obtained using the modified cellulose-based biosorbents, the practical application of these materials might be limited due to the tremendous consumption of energy and chemicals for material purification and preparation. Bacterial cellulose-based adsorbents, therefore, offer many notable advantages, including viable production, high purity, facile pre-treatment, and no required toxic chemicals.^61,70^ More importantly, in this study, it can be highlighted that the bacterial cellulose aerogel derived from the pineapple peel waste showed a comparable efficiency of 29.6 mg/g in capturing methylene blue from an aqueous solution without any further chemical functionalization (entry 15, Table 3). In addition, valorization strategies for fruit and vegetable wastes should be implemented to reduce disposal costs and environmental effects.^71^ Therefore, the utilization of pineapple peel for the preparation of bacterial cellulose for water treatment could be considered a promising sustainable approach to raise the economic value of this abundant waste.

**Table 3:** MB Adsorption Capacity of Different Cellulose-Based Materials

Entry	Material	Adsorption conditions	Adsorption capacity (mg/g)	Refs
1	Cetyltrimethylammonium bromide-modified carboxymethyl cellulose/bagasse cryogel	pH = 7, 25 °C, *C*_o_ = 5 ppm, dose = 100 mg/3 mL, 60 min	1.4	(72)
2	Oxidized cellulose nanofibers/polyvinyl alcohol/montmorillonite K-10 composite aerogel	25 °C, *C*_o_ = 20 ppm, dose = 400 mg/50 mL, 25 min	2.3	(58)
3	Hydroxypropyl cellulose/graphene oxide hydrogel	25 °C, *C*_o_ = 80 ppm, dose = 190 mg/100 mL, 10 h	11.5	(73)
4	Carboxymethyl cellulose/k-carrageenan/activated montmorillonite composite bead	pH = 6, 30 °C, *C*_o_ = 25 ppm, dose = 100 mg/50 mL, 300 min	12.4	(74)
5	Hydrolyzed wheat straw	pH = 8, 23 °C, *C*_o_ = 14 ppm, dose = 500 mg/500 mL, 280 min	16.2	(75)
6	Magnetite/phenylenediamine/cellulose acetate nanocomposite	pH = 6, 25 °C, *C*_o_ = 50 ppm, dose = 30 mg/20 mL, 70 min	29	(76)
7	Magnetic cellulose/graphene oxide composite	pH = 6, 25 °C, *C*_o_ = 30 ppm, dose = 50 mg/50 mL, 14 h	29.5	(77)
8	Porous cellulose microbead derived from waste cotton treated with NaOH/urea	pH = 7, 25 °C, *C*_o_ = 100 ppm, dose = 1 g/50 mL, 120 min	32.5	(74)
9	Sawdust-based cellulose/ZnO nanocomposite	pH = 7, 25 °C, *C*_o_ = 150 ppm, dose = 100 mg/50 mL, 300 min	42	(78)
10	Poly(acrylic acid)/nanocrystalline cellulose nanocomposite hydrogel	ambient temperature, pH = 8, *C*_o_ = 5 ppm, dose = 50 mg/100 mL, 120 min	42.2	(79)
11	Cellulose acetate/polydopamine composite nanofiber membrane	pH = 6.5, 25 °C, *C*_o_ = 50 ppm, dose = 10 mg/20 mL, 24 h	88.2	(80)
12	Sugar beet pulp cellulose/sodium alginate/iron hydroxide composite hydrogel	pH = 6.5, 25 °C, *C*_o_ = 200 ppm, dose = 30 mg/25 mL, 150 min	93	(81)
13	Carboxylated cellulose	25 °C, *C*_o_ = 35 ppm, dose = 100 mg/10 mL, 1.5 h	97.5	(82)
14	Cellulose nanofibril-based aerogel derived from sago pith waste	pH = 7, 20 °C, *C*_o_ = 90 ppm, dose = 5 mg/20 mL, 40 min	222.2	(83)
15	Bacterial cellulose aerogel derived from pineapple peel waste	pH = 6.5, 30°C, *C*_o_ = 200 ppm, dose = 20 mg/10 mL, 30 min	29.7	this study

**Figure 13:**
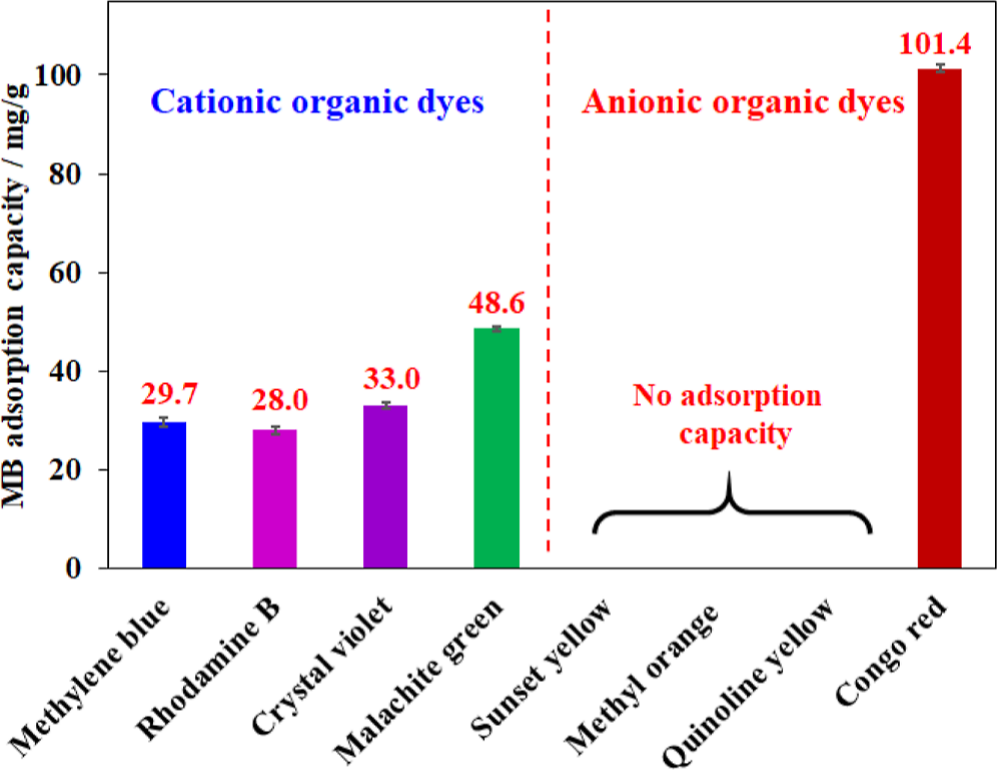
Adsorption capacity of the aerogel for various organic dyes. Adsorption condition: 20 mg of aerogel, 10 mL of dye solution with a concentration of 200 ppm, at 30 °C and pH = 6.5 for 30 min.

This study was also expanded to various cationic and anionic dyes with the results shown in Figure 13. Similar to methylene blue, other positively charged dye species derived from rhodamine B, crystal violet, and malachite green could be adsorbed by the BC aerogels due to the electron-rich oxygen atoms abundantly present in cellulose. Approximate adsorption capacities (30 mg/g) for methylene blue, rhodamine B, and crystal violet were recorded while malachite green was adsorbed with a 50% higher amount (48.6 mg/g) under identical conditions. It should be noticed that malachite green was relatively as large as crystal violet but larger than methylene blue (Table 1). Therefore, it was suggested that the removal of cationic organic dyes by the BC aerogel material was independent of their molecular size. And this process could be determined by the nature of functional groups on organic compounds, which offered various chemical interactions to adsorptive sites onto the cellulose backbone. By contrast, the BC aerogel was inefficient for the anionic species of sunset yellow, quinoline yellow, and methyl orange, while the sizes of these compounds were similar to that of methylene blue (Table 1). This could be rationalized by the inactivity or the repulsion of the electron-rich oxygen atoms onto the cellulose backbone in anionic organic dyes. Notably, an exceptional case was observed for congo red, which is also classified as an anionic organic dye. A gram of BC aerogel can adsorb up to 101.4 mg of congo red, which was significantly higher than the cationic dyes recorded in this study. It should be noted that the congo red molecule possesses two more amine groups besides the typical sulfonate moieties of the anionic dye class. Therefore, the hydrogen bonding-based interaction of these amine groups with the hydroxyl groups of cellulose could be responsible for such impressive uptake of congo red on the BC aerogel. According to Chong and co-authors, the possible formation of hydrogen bonding between hydroxyl groups of the cellulose aerogel and various hydrogen bond acceptors on the congo red backbone, including aromatic rings, nitrogen, sulfur, and oxygen atoms, could be considered as a rational reason for the impressive adsorption capacity.^84,85^ However, further studies are still needed for a better understanding of the high selectivity of aerogel materials derived from cellulose frameworks toward the congo red molecules.

To further discover the adsorption selectivity of the bacterial cellulose aerogel toward cationic and anionic dyes, an adsorption experiment was performed with a solution containing quinoline yellow (anionic dye) and methylene blue (cationic dye). The adsorption of the dyes was monitored by withdrawing aliquots from the solution at different time intervals for UV–vis spectroscopy. The initial solution exhibited two absorbance peaks at 441 and 664 nm, indicating the presence of these anionic and cationic dyes. As can be predicted, there was a significant drop in the intensity of the 664 nm peak within only 1 min of contact with the aerogel while the 441 nm peak remained almost unchanged during the 30 min experiment (Figure S3). The different adsorption behavior between MB and QY with the BC aerogel indeed proved the high affinity of the electron-rich bacterial cellulose surface with the positively charged species. On the other hand, the repulsive interaction between the aerogel and the anions was dominant, resulting in the inefficiency of trapping the anionic dyes. However, the introduction of amine groups to the dye anions, e.g., congo red, could improve the attraction toward the cellulose matrix via the formation of hydrogen bonding on the cellulose surface.

**Figure 14:**
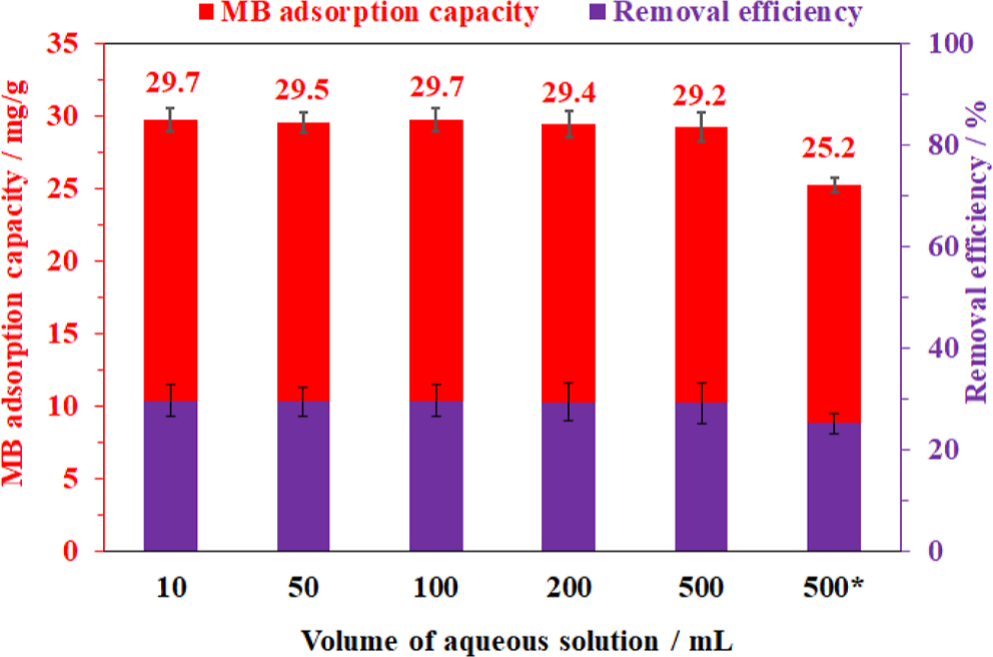
Effect of aqueous solution volume on methylene blue adsorption capacity of the aerogel and recycling test employing cellulose-based aerogel in the removal of methylene blue (*). Adsorption condition: solid/liquid ratio = 2 mg/1 mL with a concentration of 200 ppm MB, at 30 °C and pH = 6.5 for 30 min.

Notably, the adsorption for MB was scaled up to 5–50 times larger batches by increasing the adsorption volume while keeping the same ratio of adsorbent/solution. The result showed that the MB trapping capacity was in a range from 29.2 to 29.8 mg/g for all tested scales from 10 to 500 mL, indicating that there was no mass transfer limitation in the adsorption even scaling up 50 times (Figure 14). These results demonstrated that the bacterial cellulose-based material prepared in this work would be a promising ideal platform for the removal of harmful chemicals from an aqueous solution.

The recovery and reuse of adsorbents are one of the most important objectives toward zero waste in practical processes.^61^ In this study, the recyclability of the MB-saturated aerogel was carried out by washing with different solvents. The used aerogel was isolated after a 30 min adsorption experiment. Three pure single solvents, including water, ethanol, and acetone, in which MB is well soluble, were used to wash MB from the spent aerogel. However, the complete removal of MB was unsuccessful as the MB molecules were durably trapped in the BC matrix upon chemical interactions, even though a large amount of these solvents was applied (Figure S4a–c). Alternatively, based on the fact that the MB adsorption was poor under acidic conditions, an acid-containing solution, namely, 5 wt % HCl in acetone was applied. It was observed that the MB species absorbed in the aerogel were efficiently washed out with this acidic solution as the adsorbent turned back to its original white color (Figure S4d). This could be explained by the fact that the trapped cationic species were facilely exchanged with protons in the acidic solution. Moreover, a proton excess might prevent the re-adsorption of MB at the adsorptive sites of the BC backbone. The resulting solid was subsequently washed three times with water until neutralization, prior to freeze-drying to reproduce the aerogel form (Figure S5). Characterization of the regenerated aerogel by XRD, FT-IR, and SEM showed that no significant structural alterations had occurred (Figure S6). Indeed, the three-dimensional crystalline framework of bacterial cellulose in the recycled aerogel was maintained in comparison to the fresh one. However, approx. 5% of the initial aerogel mass was lost possibly due to acidic hydrolysis. The spent bacterial cellulose-based material was reused for a new cycle of the MB adsorption experiment under identical conditions at the 500 mL scale. As shown in Figure 14, the MB-trapping efficiency decreased by approx. 14%. This decrease in the adsorption capacity was partially attributed to the aerogel loss during regeneration. The result also indicated that another fraction of about 9% of the adsorption sites was not recovered through regeneration. Therefore, it would be concluded that the used aerogel from the adsorption of MB can be partially regenerated; however, complete regeneration is still challenging and requires further study.

## 4 Conclusions

In this work, *nata de pina* obtained from the bacterial fermentation of pineapple water was employed as the cost-efficient bacterial cellulose source to produce highly crystallite and ultralight aerogels via the freeze-drying technique. These aerogels have a 3D network with large pores derived from the unordered interconnection of cellulose fibers and a high density of the hydroxyl group on the surface, which could efficiently adsorb cationic dyes and a selected anionic dye. The cellulose content (0.24–0.80%) in the aerogel materials only showed a modest impact on capturing methylene blue, while the adsorption conditions, including exposure time, temperature, initial pH value, and concentration of the solution significantly affected the adsorption performance. A remarkable MB uptake of 29.7 mg/g was found at 30 °C, pH = 6.5 for 30 min. The adsorption of methylene blue onto cellulose aerogels was fast and fit well with the pseudo-second-order and Langmuir adsorption models. Besides, the aerogel derived from bacterial cellulose also exhibited remarkable selective removal of different cationic dyes, including rhodamine B, crystal violet, and malachite green, with adsorption capacities of 28.0, 33.0, and 48.6 mg/g, respectively, due to a strong interaction between the positive charge of these cation dyes with the negative charge of the hydroxyl group on the surface of the cellulose aerogels. By contrast, no uptakes were recorded for most of the anionic dyes, namely methyl orange, sunset yellow, and quinoline yellow, because of repulsion between the two negative charges of the dyes and the adsorbents. However, an exception for one anionic dye—congo red—was recorded, in which the cellulose aerogels showed a substantially high adsorption capacity (101.4 mg/g) due to a strong hydrogen bond between the cellulose aerogels and the two amine groups of red congo. More importantly, the aerogel materials exhibited the same performance in up-scaling conditions of 5–50 times.

## Supporting Information Available

FTIR spectrum of fresh cellulose-based aerogel; determination of the zero-charge point (pH_PZC_) of the cellulose-based aerogel; UV–vis absorption spectra of the mixture of quinoline yellow and methylene blue (200 ppm:200 ppm) in the presence of cellulose-based aerogel at 30 °C and pH = 6.5; photographs of the collected samples after washing three times different solvents such as water, ethanol, acetone, and acetone solution containing 5 wt % of HCl; photographs of the regenerated aerogel after freeze-drying; and characterization of the used cellulose-based aerogel after the adsorption (PDF)

## References

[ref1] Arman N. Z.; Salmiati S.; Aris A.; Salim M. R.; Nazifa T. H.; Muhamad M. S.; Marpongahtun M. A Review on Emerging Pollutants in the Water Environment: Existences, Health Effects and Treatment Processes. Water 2021, 13, 3258 10.3390/w13223258.

[ref2] Brezonik P. L.; Arnold W. A. Water Chemistry: Fifty Years of Change and Progress. Environ. Sci. Technol. 2012, 46, 5650–5657. 10.1021/es300882y. 22563943

[ref3] Aldalbahi A.; El-Naggar M. E.; El-Newehy M. H.; Rahaman M.; Hatshan M. R.; Khattab T. A. Effects of Technical Textiles and Synthetic Nanofibers on Environmental Pollution. Polymers 2021, 13, 155 10.3390/polym13010155. 33401538 PMC7794755

[ref4] Hansen É.; Monteiro de Aquim P.; Hansen A. W.; Cardoso J. K.; Ziulkoski A. L.; Gutterres M. Impact of post-tanning chemicals on the pollution load of tannery wastewater. J. Environ. Manage. 2020, 269, 110787 10.1016/j.jenvman.2020.110787. 32430280

[ref5] Hughes S. R.; Kay P.; Brown L. E. Global Synthesis and Critical Evaluation of Pharmaceutical Data Sets Collected from River Systems. Environ. Sci. Technol. 2013, 47, 661–677. 10.1021/es3030148. 23227929 PMC3636779

[ref6] Rathi B. S.; Kumar P. S.; Vo D. V. N. Critical review on hazardous pollutants in water environment: Occurrence, monitoring, fate, removal technologies and risk assessment. Sci. Total Environ. 2021, 797, 149134 10.1016/j.scitotenv.2021.149134. 34346357

[ref7] Lellis B.; Fávaro-Polonio C. Z.; Pamphile J. A.; Polonio J. C. Effects of textile dyes on health and the environment and bioremediation potential of living organisms. Biotechnol. Res. Innov. 2019, 3, 275–290. 10.1016/j.biori.2019.09.001.

[ref8] Mohammadi A.; Veisi P. High adsorption performance of β-cyclodextrin-functionalized multi-walled carbon nanotubes for the removal of organic dyes from water and industrial wastewater. J. Environ. Chem. Eng. 2018, 6, 4634–4643. 10.1016/j.jece.2018.07.002.

[ref9] Xiao W.; Jiang X.; Liu X.; Zhou W.; Garba Z. N.; Lawan I.; Wang L.; Yuan Z. Adsorption of organic dyes from wastewater by metal-doped porous carbon materials. J. Cleaner Prod. 2021, 284, 124773 10.1016/j.jclepro.2020.124773.

[ref10] Nguyen K. D.; Ho P. H.; Vu P. D.; Pham T. L. D.; Trens P.; Di Renzo F.; Phan N. T. S.; Le H. V. Efficient Removal of Chromium(VI) Anionic Species and Dye Anions from Water Using MOF-808 Materials Synthesized with the Assistance of Formic Acid. Nanomaterials 2021, 11, 1398 10.3390/nano11061398. 34070500 PMC8226478

[ref11] Hashim M. A.; Mukhopadhyay S.; Sahu J. N.; Sengupta B. Remediation technologies for heavy metal contaminated groundwater. J. Environ. Manage. 2011, 92, 2355–2388. 10.1016/j.jenvman.2011.06.009. 21708421

[ref12] Gupta V. K.; Carrott P. J. M.; Ribeiro Carrott M. M. L.; Suhas Low-Cost Adsorbents: Growing Approach to Wastewater Treatment—a Review. Crit. Rev. Environ. Sci. Technol. 2009, 39, 783–842. 10.1080/10643380801977610.

[ref13] Wolok E.; Barafi J.; Joshi N.; Girimonte R.; Chakraborty S. Study of bio-materials for removal of the oil spill. Arabian J. Geosci. 2020, 13, 1244 10.1007/s12517-020-06244-3.

[ref14] Khamkeaw A.; Jongsomjit B.; Robison J.; Phisalaphong M. Activated carbon from bacterial cellulose as an effective adsorbent for removing dye from aqueous solution. Sep. Sci. Technol. 2018, 54, 2180–2193. 10.1080/01496395.2018.1541906.

[ref15] Maleki H. Recent advances in aerogels for environmental remediation applications: A review. Chem. Eng. J. 2016, 300, 98–118. 10.1016/j.cej.2016.04.098.

[ref16] Wang Y.; Su Y.; Wang W.; Fang Y.; Riffat S. B.; Jiang F. The advances of polysaccharide-based aerogels: Preparation and potential application. Carbohydr. Polym. 2019, 226, 115242 10.1016/j.carbpol.2019.115242. 31582065

[ref17] Chen X.; Cui J.; Xu X.; Sun B.; Zhang L.; Dong W.; Chen C.; Sun D. Bacterial cellulose/attapulgite magnetic composites as an efficient adsorbent for heavy metal ions and dye treatment. Carbohydr. Polym. 2020, 229, 115512 10.1016/j.carbpol.2019.115512. 31826502

[ref18] Budtova T. Cellulose II aerogels: a review. Cellulose 2019, 26, 81–121. 10.1007/s10570-018-2189-1.

[ref19] Galiwango E.; Abdel Rahman N. S.; Al-Marzouqi A. H.; Abu-Omar M. M.; Khaleel A. A. Isolation and characterization of cellulose and α-cellulose from date palm biomass waste. Heliyon 2019, 5, e02937 10.1016/j.heliyon.2019.e02937. 32382665 PMC7201136

[ref20] Wang N.; Xu B.; Wang X.; Lang J.; Zhang H. Chemical and Structural Elucidation of Lignin and Cellulose Isolated Using DES from Bagasse Based on Alkaline and Hydrothermal Pretreatment. Polymers 2022, 14, 2756 10.3390/polym14142756. 35890532 PMC9325185

[ref21] Rehman N.; Alam S.; Amin N. U.; Mian I.; Ullah H. Ecofriendly Isolation of Cellulose from Eucalyptus lenceolata: A Novel Approach. Int. J. Polym. Sci. 2018, 2018, 8381501–7. 10.1155/2018/8381501.

[ref22] Urbina L.; Corcuera M. Á.; Gabilondo N.; Eceiza A.; Retegi A. A review of bacterial cellulose: sustainable production from agricultural waste and applications in various fields. Cellulose 2021, 28, 8229–8253. 10.1007/s10570-021-04020-4.

[ref23] Anwar B.; Bundjali B.; Arcana I. M. Isolation of Cellulose Nanocrystals from Bacterial Cellulose Produced from Pineapple Peel Waste Juice as Culture Medium. Procedia Chem. 2015, 16, 279–284. 10.1016/j.proche.2015.12.051.

[ref24] Raghav N.; Sharma M. R.; Kennedy J. F. Nanocellulose: A mini-review on types and use in drug delivery systems. Carbohydr. Polym. Technol. Appl. 2021, 2, 100031 10.1016/j.carpta.2020.100031.

[ref25] Iguchi M.; Yamanaka S.; Budhiono A. Bacterial cellulose—a masterpiece of nature’s arts. J. Mater. Sci. 2000, 35, 261–270. 10.1023/a:1004775229149.

[ref26] Fleury B.; Abraham E.; De La Cruz J. A.; Chandrasekar V. S.; Senyuk B.; Liu Q.; Cherpak V.; Park S.; ten Hove J. B.; Smalyukh I. I. Aerogel from Sustainably Grown Bacterial Cellulose Pellicles as a Thermally Insulative Film for Building Envelopes. ACS Appl. Mater. Interfaces 2020, 12, 34115–34121. 10.1021/acsami.0c08879. 32615033

[ref27] Chen Y.; Zhang L.; Yang Y.; Pang B.; Xu W.; Duan G.; Jiang S.; Zhang K. Recent Progress on Nanocellulose Aerogels: Preparation, Modification, Composite Fabrication, Applications. Adv. Mater. 2021, 33, 2005569 10.1002/adma.202005569. PMC11468492 33538067

[ref28] Huang Y.; Huang X.; Ma M.; Hu C.; Seidi F.; Yin S.; Xiao H. Recent advances on the bacterial cellulose-derived carbon aerogels. J. Mater. Chem. C 2021, 9, 818–828. 10.1039/d0tc05433j.

[ref29] Pham T. T.; Tran T. T. A. Evaluation of the crystallinity of bacterial cellulose produced from pineapple waste solution by using acetobacter xylinum. ASEAN Eng. J. 2023, 13, 81–91. 10.11113/aej.v13.18868.

[ref30] Feng J.; Nguyen S. T.; Fan Z.; Duong H. M. Advanced fabrication and oil absorption properties of super-hydrophobic recycled cellulose aerogels. Chem. Eng. J. 2015, 270, 168–175. 10.1016/j.cej.2015.02.034.

[ref31] Eichhorn S. J.; Sampson W. W. Relationships between specific surface area and pore size in electrospun polymer fibre networks. J. R. Soc., Interface 2010, 7, 641–649. 10.1098/rsif.2009.0374. 19812071 PMC2842785

[ref32] Cervin N. T.; Aulin C.; Larsson P. T.; Wågberg L. Ultra porous nanocellulose aerogels as separation medium for mixtures of oil/water liquids. Cellulose 2012, 19, 401–410. 10.1007/s10570-011-9629-5.

[ref33] Revellame E. D.; Fortela D. L.; Sharp W.; Hernandez R.; Zappi M. E. Adsorption kinetic modeling using pseudo-first order and pseudo-second order rate laws: A review. Clean. Eng. and Technol. 2020, 1, 100032 10.1016/j.clet.2020.100032.

[ref34] Kalam S.; Abu-Khamsin S. A.; Kamal M. S.; Patil S. Surfactant Adsorption Isotherms: A Review. ACS Omega 2021, 6, 32342–32348. 10.1021/acsomega.1c04661. 34901587 PMC8655760

[ref35] Mullet M.; Fievet P.; Szymczyk A.; Foissy A.; Reggiani J. C.; Pagetti J. A simple and accurate determination of the point of zero charge of ceramic membranes. Desalination 1999, 121, 41–48. 10.1016/s0011-9164(99)00006-5.

[ref36] Abral H.; Lawrensius V.; Handayani D.; Sugiarti E. Preparation of nano-sized particles from bacterial cellulose using ultrasonication and their characterization. Carbohydr. Polym. 2018, 191, 161–167. 10.1016/j.carbpol.2018.03.026. 29661304

[ref37] Li Z.; Zhong L.; Zhang T.; Qiu F.; Yue X.; Yang D. Sustainable, Flexible, and Superhydrophobic Functionalized Cellulose Aerogel for Selective and Versatile Oil/Water Separation. ACS Sustain. Chem. Eng. 2019, 7, 9984–9994. 10.1021/acssuschemeng.9b01122.

[ref38] Mohamed M. A.; Abd Mutalib M.; Mohd Hir Z. A.; M Zain M.; Mohamad A. B.; Jeffery Minggu L.; Awang N. A.; Salleh W.; Salleh W. N. An overview on cellulose-based material in tailoring bio-hybrid nanostructured photocatalysts for water treatment and renewable energy applications. Int. J. Biol. Macromol. 2017, 103, 1232–1256. 10.1016/j.ijbiomac.2017.05.181. 28587962

[ref39] Mohamad S.; Abdullah L. C.; Jamari S. S.; Al Edrus S. S. O.; Aung M. M.; Mohamad S. F. S. Influence of drying method on the crystal structure and thermal property of oil palm frond juice-based bacterial cellulose. J. Mater. Sci. 2022, 57, 1462–1473. 10.1007/s10853-021-06685-5.

[ref40] Illa M. P.; Sharma C. S.; Khandelwal M. Tuning the physiochemical properties of bacterial cellulose: effect of drying conditions. J. Mater. Sci. 2019, 54, 12024–12035. 10.1007/s10853-019-03737-9.

[ref41] Ul-Islam M.; Khattak W. A.; Kang M.; Kim S. M.; Khan T.; Park J. K. Effect of post-synthetic processing conditions on structural variations and applications of bacterial cellulose. Cellulose 2013, 20, 253–263. 10.1007/s10570-012-9799-9.

[ref42] Zhang C. J.; Wang L.; Zhao J. C.; Zhu P. Effect of Drying Methods on Structure and Mechanical Properties of Bacterial Cellulose Films. Adv. Mater. Res. 2011, 239–242, 2667–2670. 10.4028/www.scientific.net/amr.239-242.2667.

[ref43] Zimmermann M. V. G.; Borsoi C.; Lavoratti A.; Zanini M.; Zattera A. J.; Santana R. M. C. Drying techniques applied to cellulose nanofibers. J. Reinf. Plast. Compos. 2016, 35, 682–697. 10.1177/0731684415626286.

[ref44] Liebner F.; Potthast A.; Rosenau T.; Haimer E.; Wendland M. Cellulose aerogels: Highly porous, ultra-lightweight materials. Holzforschung 2008, 62, 129–135. 10.1515/hf.2008.051.

[ref45] Liebner F.; Haimer E.; Potthast A.; Loidl D.; Tschegg S.; Neouze M.-A.; Wendland M.; Rosenau T. Cellulosic aerogels as ultra-lightweight materials. Part 2: Synthesis and properties 2^nd^ ICC 2007, Tokyo, Japan, October 25–29, 2007. Holzforschung 2009, 63, 3–11. 10.1515/hf.2009.002.

[ref46] Liebner F.; Haimer E.; Wendland M.; Neouze M. A.; Schlufter K.; Miethe P.; Heinze T.; Potthast A.; Rosenau T. Aerogels from unaltered bacterial cellulose: application of scCO2 drying for the preparation of shaped, ultra-lightweight cellulosic aerogels. Macromol. Biosci. 2010, 10, 349–352. 10.1002/mabi.200900371. 20166232

[ref47] Chang S. S.; Clair B.; Ruelle J.; Beauchene J.; Di Renzo F.; Quignard F.; Zhao G. J.; Yamamoto H.; Gril J. Mesoporosity as a new parameter for understanding tension stress generation in trees. J. Exp. Bot. 2009, 60, 3023–3030. 10.1093/jxb/erp133. 19436045

[ref48] Guo J.; Catchmark J. M. Surface area and porosity of acid hydrolyzed cellulose nanowhiskers and cellulose produced by Gluconacetobacter xylinus. Carbohydr. Polym. 2012, 87, 1026–1037. 10.1016/j.carbpol.2011.07.060.

[ref49] Horvat G.; Pantic M.; Knez Z.; Novak Z. A Brief Evaluation of Pore Structure Determination for Bioaerogels. Gels 2022, 8, 438 10.3390/gels8070438. 35877523 PMC9316429

[ref50] Mi Q.-y.; Ma S.-r.; Yu J.; He J.-s.; Zhang J. Flexible and Transparent Cellulose Aerogels with Uniform Nanoporous Structure by a Controlled Regeneration Process. ACS Sustain. Chem. Eng. 2016, 4, 656–660. 10.1021/acssuschemeng.5b01079.

[ref51] Dos Santos Silva L.; De Oliveira Carvalho J.; De Sousa Bezerra R. D.; Da Silva M. S.; Ferreira F. J.; Osajima J. A.; Da Silva Filho E. C. Potential of Cellulose Functionalized with Carboxylic Acid as Biosorbent for the Removal of Cationic Dyes in Aqueous Solution. Molecules 2018, 23, 743 10.3390/molecules23040743. 29570648 PMC6017135

[ref52] Lahiri D.; Nag M.; Dutta B.; Dey A.; Sarkar T.; Pati S.; Edinur H. A.; Abdul Kari Z.; Mohd Noor N. H.; Ray R. R. Bacterial Cellulose: Production, Characterization, and Application as Antimicrobial Agent. Int. J. Mol. Sci. 2021, 22, 12984 10.3390/ijms222312984. 34884787 PMC8657668

[ref53] Yang H.; Yan R.; Chen H.; Lee D. H.; Zheng C. Characteristics of hemicellulose, cellulose and lignin pyrolysis. Fuel 2007, 86, 1781–1788. 10.1016/j.fuel.2006.12.013.

[ref54] Eltaweil A. S.; Elgarhy G. S.; El-Subruiti G. M.; Omer A. M. Carboxymethyl cellulose/carboxylated graphene oxide composite microbeads for efficient adsorption of cationic methylene blue dye. Int. J. Biol. Macromol. 2020, 154, 307–318. 10.1016/j.ijbiomac.2020.03.122. 32184142

[ref55] Chan C. H.; Chia C. H.; Zakaria S.; Sajab M. S.; Chin S. X. Cellulose nanofibrils: a rapid adsorbent for the removal of methylene blue. RSC Adv. 2015, 5, 18204–18212. 10.1039/c4ra15754k.

[ref56] Nguyen V. T.; Ha L. Q.; Nguyen T. D. L.; Ly P. H.; Nguyen D. M.; Hoang D. Nanocellulose and Graphene Oxide Aerogels for Adsorption and Removal Methylene Blue from an Aqueous Environment. ACS Omega 2022, 7, 1003–1013. 10.1021/acsomega.1c05586. 35036764 PMC8756800

[ref57] Huang J.; Yan Z. Adsorption Mechanism of Oil by Resilient Graphene Aerogels from Oil–Water Emulsion. Langmuir 2018, 34, 1890–1898. 10.1021/acs.langmuir.7b03866. 29307185

[ref58] Luo M.; Wang M.; Pang H.; Zhang R.; Huang J.; Liang K.; Chen P.; Sun P.; Kong B. Super-assembled highly compressible and flexible cellulose aerogels for methylene blue removal from water. Chin. Chem. Lett. 2021, 32, 2091–2096. 10.1016/j.cclet.2021.03.024.

[ref59] Phuong N. T. X.; Ho K. H.; Nguyen C. T. X.; Dang Y. T.; Do N. H. N.; Le K. A.; Do T. C. Novel Fabrication of Renewable Aerogels from Coconut Coir Fibers for Dye Removal. Chem. Eng. Trans. 2021, 89, 31 10.3303/CET2189006.

[ref60] Hosseini H.; Zirakjou A.; McClements D. J.; Goodarzi V.; Chen W.-H. Removal of methylene blue from wastewater using ternary nanocomposite aerogel systems: Carboxymethyl cellulose grafted by polyacrylic acid and decorated with graphene oxide. J. Hazard. Mater. 2022, 421, 126752 10.1016/j.jhazmat.2021.126752. 34352524

[ref61] Ahmad T.; Danish M.; Rafatullah M.; Ghazali A.; Sulaiman O.; Hashim R.; Ibrahim M. N. M. The use of date palm as a potential adsorbent for wastewater treatment: a review. Environ. Sci. Pollut. Res. 2012, 19, 1464–1484. 10.1007/s11356-011-0709-8. 22207239

[ref62] Nguyen K. D.; Vo N. T.; Le K. T. M.; Ho K. V.; Phan N. T. S.; Ho P. H.; Le H. V. Defect-engineered metal–organic frameworks (MOF-808) towards the improved adsorptive removal of organic dyes and chromium (vi) species from water. New J. Chem. 2023, 47, 6433–6447. 10.1039/d2nj05693c.

[ref63] Elsayed I.; Schueneman G. T.; El-Giar E. M.; Hassan E. B. Amino-Functionalized Cellulose Nanofiber/Lignosulfonate New Aerogel Adsorbent for the Removal of Dyes and Heavy Metals from Wastewater. Gels 2023, 9, 154 10.3390/gels9020154. 36826324 PMC9956574

[ref64] Salazar-Rabago J. J.; Leyva-Ramos R.; Rivera-Utrilla J.; Ocampo-Perez R.; Cerino-Cordova F. J. Biosorption mechanism of Methylene Blue from aqueous solution onto White Pine (Pinus durangensis) sawdust: Effect of operating conditions. Sustainable Environ. Res. 2017, 27, 32–40. 10.1016/j.serj.2016.11.009.

[ref65] Bialik E.; Stenqvist B.; Fang Y.; Östlund Å.; Furó I.; Lindman B.; Lund M.; Bernin D. Ionization of Cellobiose in Aqueous Alkali and the Mechanism of Cellulose Dissolution. J. Phys. Chem. Lett. 2016, 7, 5044–5048. 10.1021/acs.jpclett.6b02346. 27973886

[ref66] Swensson B.; Larsson A.; Hasani M. Probing Interactions in Combined Hydroxide Base Solvents for Improving Dissolution of Cellulose. Polymers 2020, 12, 1310 10.3390/polym12061310. 32521817 PMC7362248

[ref67] Wei X.; Huang T.; Nie J.; Yang J.-h.; Qi X.-d.; Zhou Z.-w.; Wang Y. Bio-inspired functionalization of microcrystalline cellulose aerogel with high adsorption performance toward dyes. Carbohydr. Polym. 2018, 198, 546–555. 10.1016/j.carbpol.2018.06.112. 30093033

[ref68] Wan C.; Zhang L.; Yong K.-T.; Li J.; Wu Y. Recent progress in flexible nanocellulosic structures for wearable piezoresistive strain sensors. J. Mater. Chem. C 2021, 9, 11001–11029. 10.1039/d1tc02360h.

[ref69] Wang S.; Zhang Q.; Wang Z.; Pu J. Facile fabrication of an effective nanocellulose-based aerogel and removal of methylene blue from aqueous system. J. Water Process Eng. 2020, 37, 101511 10.1016/j.jwpe.2020.101511.

[ref70] Nguyen H. H. M.; Tan K. V. M.; Van T. T. T.; Nguyen H. N.; Phan A. N. Q.; Tran A. T. T.; Le P. K.; Le K. A.; Nguyen K. D.; Le H. V. Preparation of Cu-modified bacterial cellulose aerogels derived from nata de coco towards the enhanced adsorption of hydrophobic organic solvents. J. Porous Mater. 2023, 30, 1195–1205. 10.1007/s10934-022-01413-z.

[ref71] Nath P. C.; Ojha A.; Debnath S.; Neetu K.; Bardhan S.; Mitra P.; Sharma M.; Sridhar K.; Nayak P. K. Recent advances in valorization of pineapple (Ananas comosus) processing waste and by-products: A step towards circular bioeconomy. Trends Food Sci. Technol. 2023, 136, 100–111. 10.1016/j.tifs.2023.04.008.

[ref72] Meneses I. P.; Novaes S. D.; Dezotti R. S.; Oliveira P. V.; Petri D. F. S. CTAB-modified carboxymethyl cellulose/bagasse cryogels for the efficient removal of bisphenol A, methylene blue and Cr(VI) ions: Batch and column adsorption studies. J. Hazard. Mater. 2022, 421, 126804 10.1016/j.jhazmat.2021.126804. 34388928

[ref73] Liu X.; Zhou Y.; Nie W.; Song L.; Chen P. Fabrication of hydrogel of hydroxypropyl cellulose (HPC) composited with graphene oxide and its application for methylene blue removal. J. Mater. Sci. 2015, 50, 6113–6123. 10.1007/s10853-015-9166-y.

[ref74] Hua J.; Meng R.; Wang T.; Gao H.; Luo Z.; Jin Y.; Liu L.; Yao J. Highly Porous Cellulose Microbeads and their Adsorption for Methylene Blue. Fibers Polym. 2019, 20, 794–803. 10.1007/s12221-019-8334-0.

[ref75] Batzias F.; Sidiras D.; Schroeder E.; Weber C. Simulation of dye adsorption on hydrolyzed wheat straw in batch and fixed-bed systems. Chem. Eng. J. 2009, 148, 459–472. 10.1016/j.cej.2008.09.025.

[ref76] Mahmoud M. E.; Abdelwahab M. S. Fabricated and functionalized magnetite/phenylenediamine/cellulose acetate nanocomposite for adsorptive removal of methylene blue. Int. J. Biol. Macromol. 2019, 128, 196–203. 10.1016/j.ijbiomac.2019.01.102. 30682477

[ref77] Shi H.; Li W.; Zhong L.; Xu C. Methylene Blue Adsorption from Aqueous Solution by Magnetic Cellulose/Graphene Oxide Composite: Equilibrium, Kinetics, and Thermodynamics. Ind. Eng. Chem. Res. 2014, 53, 1108–1118. 10.1021/ie4027154.

[ref78] Oyewo O. A.; Adeniyi A.; Sithole B. B.; Onyango M. S. Sawdust-Based Cellulose Nanocrystals Incorporated with ZnO Nanoparticles as Efficient Adsorption Media in the Removal of Methylene Blue Dye. ACS Omega 2020, 5, 18798–18807. 10.1021/acsomega.0c01924. 32775881 PMC7408268

[ref79] Safavi-Mirmahalleh S.-A.; Salami-Kalajahi M.; Roghani-Mamaqani H. Effect of surface chemistry and content of nanocrystalline cellulose on removal of methylene blue from wastewater by poly(acrylic acid)/nanocrystalline cellulose nanocomposite hydrogels. Cellulose 2019, 26, 5603–5619. 10.1007/s10570-019-02490-1.

[ref80] Cheng J.; Zhan C.; Wu J.; Cui Z.; Si J.; Wang Q.; Peng X.; Turng L.-S. Highly Efficient Removal of Methylene Blue Dye from an Aqueous Solution Using Cellulose Acetate Nanofibrous Membranes Modified by Polydopamine. ACS Omega 2020, 5, 5389–5400. 10.1021/acsomega.9b04425. 32201829 PMC7081408

[ref81] Fang Y.; Liu Q.; Zhu S. Selective biosorption mechanism of methylene blue by a novel and reusable sugar beet pulp cellulose/sodium alginate/iron hydroxide composite hydrogel. Int. J. Biol. Macromol. 2021, 188, 993–1002. 10.1016/j.ijbiomac.2021.07.192. 34358601

[ref82] Cao X.; Liu M.; Bi W.; Lin J.; Chen D. D. Y. Direct carboxylation of cellulose in deep eutectic solvent and its adsorption behavior of methylene blue. Carbohydr. Polym. Technol. Appl. 2022, 4, 100222 10.1016/j.carpta.2022.100222.

[ref83] Beh J. H.; Lim T. H.; Lew J. H.; Lai J. C. Cellulose nanofibril-based aerogel derived from sago pith waste and its application on methylene blue removal. Int. J. Biol. Macromol. 2020, 160, 836–845. 10.1016/j.ijbiomac.2020.05.227. 32485260

[ref84] Chong K. Y.; Chia C. H.; Zakaria S.; Sajab M. S.; Chook S. W.; Khiew P. S. CaCO3-decorated cellulose aerogel for removal of Congo Red from aqueous solution. Cellulose 2015, 22, 2683–2691. 10.1007/s10570-015-0675-2.

[ref85] Abramian L.; El-Rassy H. Adsorption kinetics and thermodynamics of azo-dye Orange II onto highly porous titania aerogel. Chem. Eng. J. 2009, 150, 403–410. 10.1016/j.cej.2009.01.019.

