# Supplementary material for: Bacterial Cellulose Aerogels Derived from Pineapple Peel Waste for the Adsorption of Dyes
Source: ACS Omega. 2023 Sep 1;8(37):33412–25. doi: 10.1021/acsomega.3c03130 (PMC10515182; doi:10.1021/acsomega.3c03130)
Supplement: Supplementary file 1 — ao3c03130_si_001.pdf [file ao3c03130_si_001.pdf]

Supporting Information

Bacterial cellulose aerogels derived from pineapple peel waste for the adsorption of dyes

Ha Vu Le,[1,2] Nghia Thi Dao,[1,2] Ha Truc Bui,[1,2] Phung Thi Kim Le, [1,2] Kien Anh Le,[3] An Thi Tuong Tran,[1,2] Khoa Dang Nguyen,[1,2,*] Hanh Huynh Mai Nguyen,[1,2,*] Phuoc Hoang Ho[4,*]

[1] Faculty of Chemical Engineering, Ho Chi Minh City University of Technology (HCMUT), 268 Ly Thuong Kiet Street, District 10, Ho Chi Minh City 740010, Vietnam

[2] Vietnam National University Ho Chi Minh City, Linh Trung Ward, Thu Duc District, Ho Chi Minh City 740010, Vietnam

[3] Institute for Tropical Technology and Environmental Protection, 57A Truong Quoc Dung Street, Phu Nhuan District, Ho Chi Minh City, Vietnam

[4] Chemical Engineering, Competence Centre for Catalysis, Chalmers University of Technology, Gothenburg, SE-412 96, Sweden.

* Corresponding author: nhmhanh.sdh21@hcmut.edu.vn (Hanh Huynh Mai Nguyen), khoand1989@hcmut.edu.vn (Khoa Dang Nguyen), phuoc@chalmers.se (Phuoc Hoang Ho)

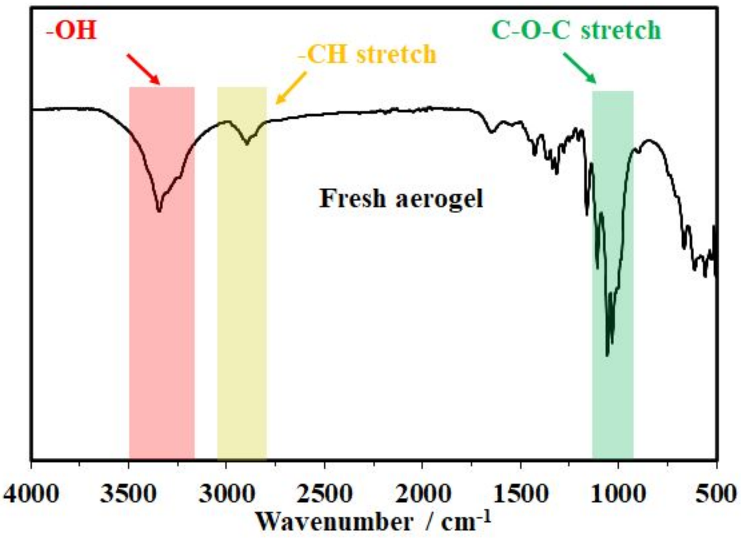

Figure S1. FT-IR spectrum of the fresh cellulose-based aerogel.

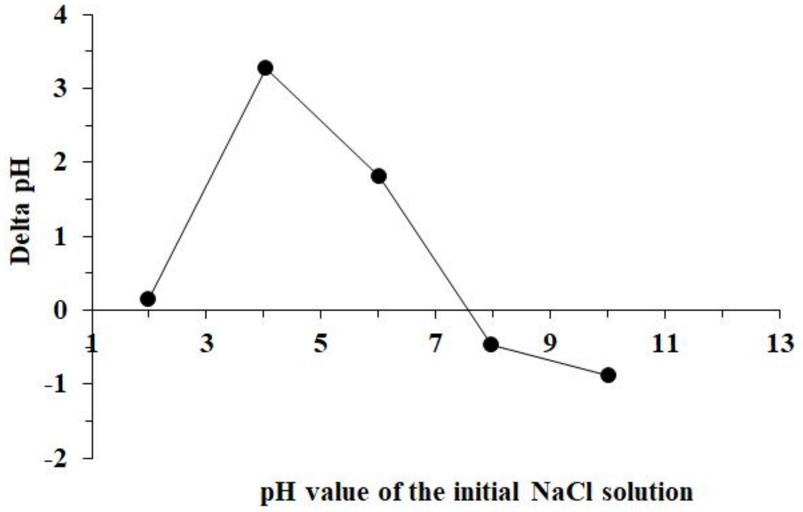

Figure S2. Determination of the zero-charge point (pH_{PZC}) of the cellulose-based aerogel.

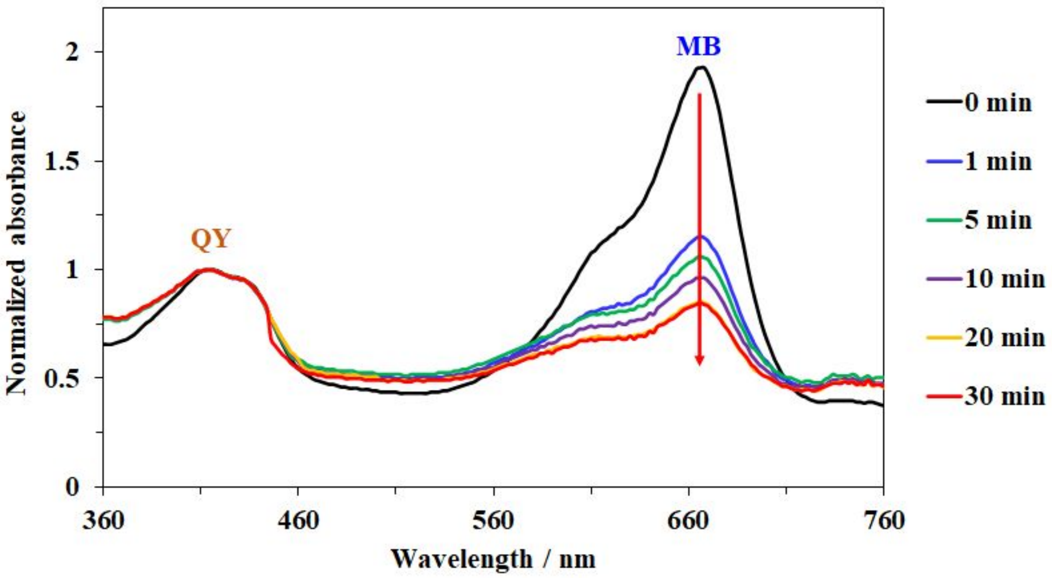

Figure S3. UV-Vis absorption spectra of the mixture of quinoline yellow (QY) and methylene blue (MB) (200 ppm : 200 ppm) in the presence of cellulose-based aerogel at 30 ^{o}C and pH = 6.5.

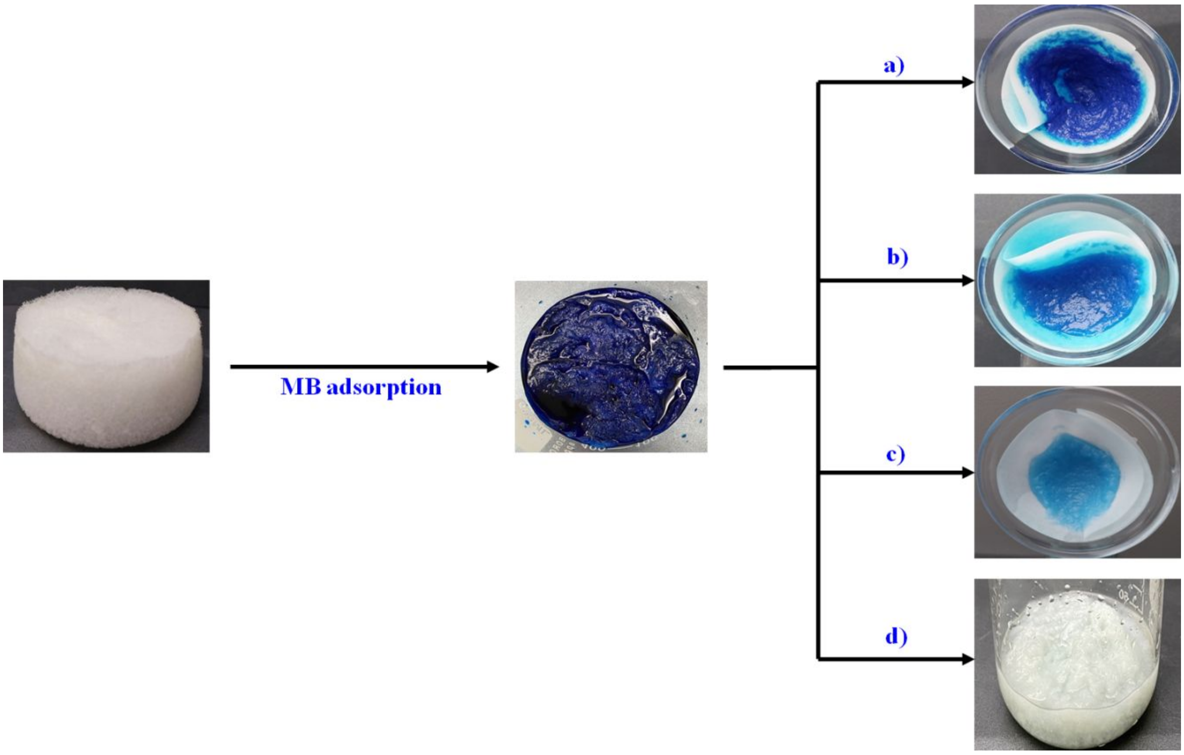

Figure S4. Photographs of the fresh and the collected samples after washing with different solvents (thrice for each) including water (a); ethanol (b); acetone (c); and acetone solution containing 5 wt.% of HCl (d).

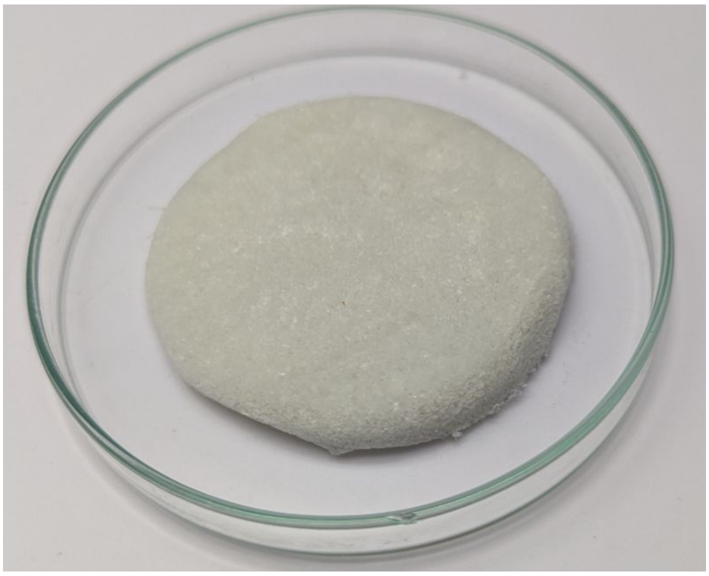

Figure S5. Photographs of the regenerated aerogel after freeze-drying.

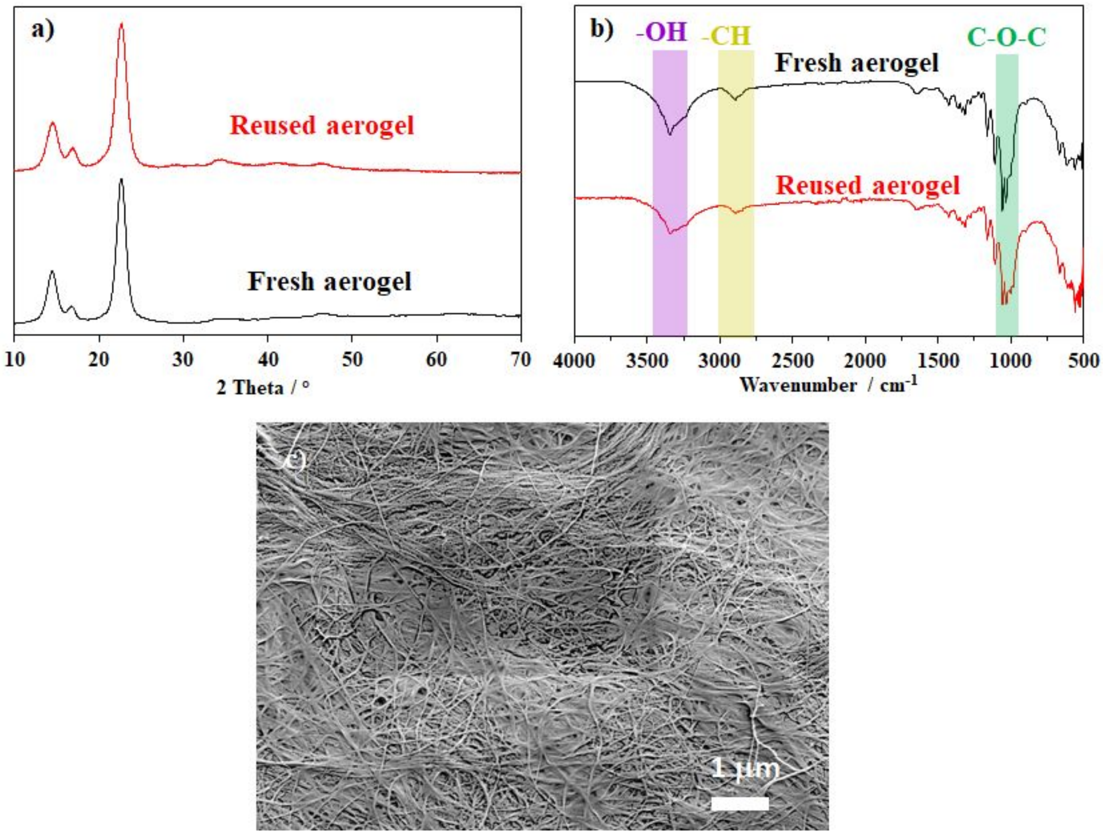

Figure S6. XRD profiles (a), and FT-IR spectra (b) of fresh and reused cellulose-based aerogel after adsorption process; and (c) SEM picture of the regenerated cellulose-based aerogel.
